# Pan‐cancer analyses reveal molecular and clinical characteristics of cuproptosis regulators

**DOI:** 10.1002/imt2.68

**Published:** 2022-12-07

**Authors:** Changwu Wu, Jun Tan, Xiangyu Wang, Chaoying Qin, Wenyong Long, Yimin Pan, Yuzhe Li, Qing Liu

**Affiliations:** ^1^ Department of Neurosurgery, Xiangya Hospital Central‐South University Changsha Hunan China; ^2^ Department of Neurosurgery, Devision of Experimental Neurosurgery University of Heidelberg Heidelberg Germany; ^3^ Institute of Skull Base Surgery and Neuro‐oncology at Hunan Changsha Hunan China

**Keywords:** cuproptosis, drug sensitivity, pan‐cancer, prognosis, tumor microenvironment, tumor‐related pathways

## Abstract

Imbalance in copper homeostasis can be lethal. A recent study found that excess copper induces cell death in a way that has never been characterized before, which is dependent on mitochondrial stress and is referred to as “cuproptosis.” The role of cuproptosis in tumors has not yet been elucidated. In this study, we revealed the complex and important roles of cuproptosis regulators and cuproptosis activity in tumors via a comprehensive analysis of multiomics data from more than 10,000 samples of 33 tumor types. We found that the cyclin‐dependent kinase inhibitor 2A is the most frequently altered cuproptosis regulator, and the cuproptosis regulator expression is dysregulated in various tumors. Additionally, we developed a cuproptosis activity score to reflect the overall cuproptosis level. On the basis of the expression levels of cuproptosis regulators, tumors can be divided into two clusters with different cuproptosis activities and survival outcomes. Importantly, cuproptosis activity was found to be associated with the prognosis of multiple tumors and multiple tumor‐related pathways, including fatty acid metabolism and remodeling of the tumor microenvironment. Furthermore, cuproptosis increased the sensitivity to multiple drugs and exhibited potential to predict the outcome of immunotherapy. We also comprehensively identified cuproptosis‐related microRNAs, long noncoding RNAs, and transcription factors. We provided the code corresponding to the results of this study in GitHub (https://github.com/Changwuuu/Cuproptosis-pancancer.git) for reference. In summary, this study reveals important molecular and clinical characteristics of cuproptosis regulators and cuproptosis activity in tumors, and suggests the use of cuproptosis as a promising tumor therapeutic approach. This study provides an important reference point for future cuproptosis‐related research.

## Highlights

- We developed a cuproptosis activity score to reflect the overall cuproptosis level, which could provide a methodological reference for future cuproptosis studies.
- Cuproptosis is associated with a variety of tumor‐related pathways, and in particular, tumors with high fatty acid metabolism may be more sensitive to cuproptosis.
- Cuproptosis is associated with the regulation of the tumor microenvironment and can predict the outcome of immunotherapy patients based on cuproptosis activity.
- This study identified cuproptosis‐related microRNAs, long noncoding RNAs, and transcription factors in each tumor type, which can provide a resource and reference for future cuproptosis‐related research.

## Graphical abstract

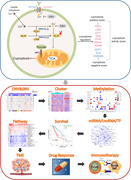

## INTRODUCTION

Transition metals are essential for complex biochemical reactions in the human body [1]. Copper (Cu), a trace metal element, is important for the maintenance of normal cellular biological functions [2, 3]. Cu is also involved in various cellular processes closely related to cell fate, such as oxidative phosphorylation, aerobic respiration, and cell growth and development [3, 4, 5, 6, 7, 8]. Maintaining balanced Cu homeostasis is critical to the body, and even small changes in Cu homeostasis can cause irreversible and serious damage [1, 9]. The most representative diseases caused by dysregulation of Cu metabolism are Wilson's disease, caused by the excessive accumulation of Cu in the liver due to defects in the transporter protein, ATPase copper transporting beta [9], and Menkes' disease, caused by severe Cu deficiency resulting from difficulties in Cu release from the enterocyte to the bloodstream due to mutations in ATPase copper transporting beta [9, 10, 11]. In addition, the role of Cu in cancer has attracted significant attention in research. Many studies have reported the accumulation of Cu in the serum samples of patients with cancer, including breast cancer [12], lung cancer [13], and hepatocellular carcinoma [14]. Mouse‐based models of hepatocellular carcinoma have also demonstrated significantly elevated levels of Cu in tumor tissues [15]. Although there is a common tendency for Cu accumulation in tumors, the association between Cu levels and cancer risk is unknown [7]. However, Cu has been shown to be important for tumor proliferation and angiogenesis [16, 17], which may explain the enrichment of Cu in tumor tissue regions. Accumulation of Cu is observed in the nuclear region of cancer cells [18]. On the basis of this, Cu chelators have been shown to inhibit tumor growth and angiogenesis [17]. For example, trientine significantly inhibits tumor development in human hepatocellular carcinoma cell lines [19], while tetrathiomolybdate inhibits tumor growth in melanoma cell lines resistant to *BRAF* or *MEK1/2* inhibitors [20].

Excessive Cu is toxic to cells [21], which explains the use of Cu ionophores to increase the Cu content and induce apoptosis of cancer cells. The anticancer effects of Cu ionophores have been demonstrated in several studies [17]. Inhibition of inflammatory breast cancer by disulfiram was confirmed both in vitro and in vivo [22], and the synergistic effects of disulfiram and docosahexaenoic acid effectively inhibited the tumor growth and promoted the apoptosis of cancer cells [23]. The cytotoxic mechanisms of metal ions, including Cu, are suggested to mainly rely on oxidative stress [24]. Oxidative stress is caused by an increase in the levels of reactive oxygen species (ROS) or highly toxic hydroxyl radicals beyond the antioxidant capacity of the cell [25]. However, a recent study published in *Science* challenges this conventional view [26]. That study revealed that intracellular Cu accumulation can induce a novel regulatory cell death mechanism via the aggregation of lipoylated mitochondrial enzymes and the destabilization of the Fe–S cluster proteins, which is termed as “cuproptosis” [26, 27]. This unique type of cell death is not affected by the levels of ROS and is different from all other oxidative stress‐related cell death mechanisms, including ferroptosis [26, 27]. Importantly, it was also found that tumor cells dependent on galactose‐mediated mitochondrial respiration were nearly 1000‐fold times more sensitive to cuproptosis than cells dependent on glucose‐induced glycolysis [26, 27]. Given that glycolysis is critical for tumor cell growth and metabolism, inhibition of glycolysis would not only suppress tumor malignancy, but also enhance sensitivity to cuproptosis, implying that the regulation of cuproptosis in tumor cells may be synergistic with other therapeutic modalities and can be potentially used to develop novel therapeutic strategies [27]. In addition, to identify the genes involved in cuproptosis, Tsvetkov et al. performed a genome‐wide CRISPR‐Cas9 loss‐of‐function screening and identified 10 genes that can regulate cuproptosis [26]. Given their regulatory role in cuproptosis activity, we refer to them as cuproptosis regulators. Among them, seven cuproptosis regulators were found to be involved in the positive regulation of cuproptosis, namely, ferredoxin 1 (*FDX1*), lipoyl synthase (*LIAS*), lipolytransferase 1 (*LIPT1*), dihydrolipoamide dehydrogenase (*DLD*), dihydrolipoamide *S*‐acetyltransferase (*DLAT*), pyruvate dehydrogenase E1 subunit *α*1 (*PDHA1*), and pyruvate dehydrogenase E1 subunit *β* (*PDHB*) [26]. Meanwhile, three of them were found to be involved in the negative regulation of cuproptosis, namely, the metal regulatory transcription factor 1 (*MTF1*), glutaminase (*GLS*), and cyclin‐dependent kinase inhibitor 2A (*CDKN2A*) [26]. The identification of these cuproptosis regulators facilitated the subsequent exploration of the mechanisms underlying Cu toxicity and regulation of this new type of cell death.

**Figure 1:**
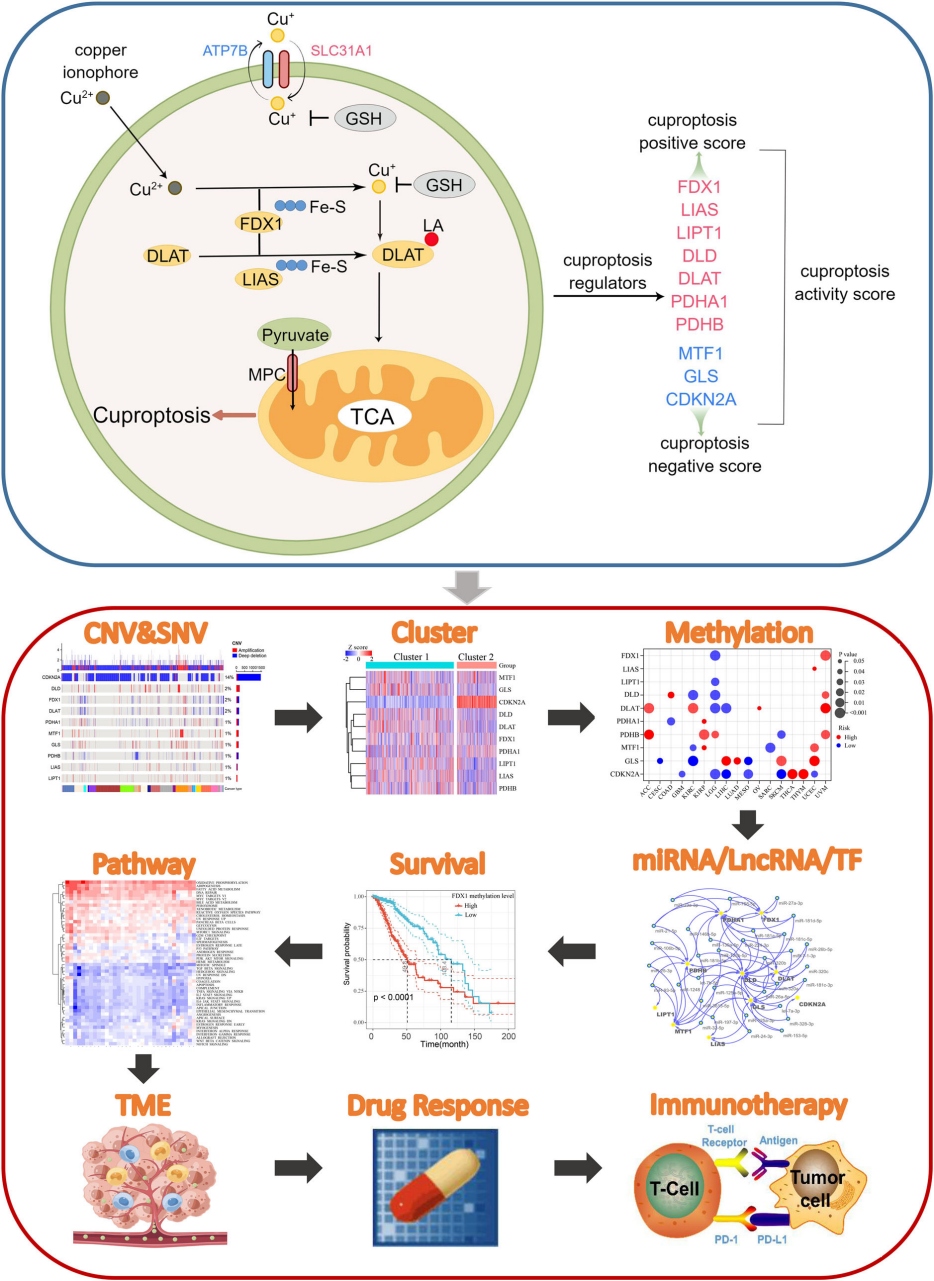
Cell model diagram of cuproptosis and the workflow of this study. CDKN2A, cyclin‐dependent kinase inhibitor 2A; CNV, copy number variation; DLAT, dihydrolipoamide *S*‐acetyltransferase; DLD, dihydrolipoamide dehydrogenase; FDX1, ferredoxin 1; GLS, glutaminase; GSH, glutathione; LIAS, lipoyl synthase; LIPT1, lipolytransferase 1; MPC, mitochondrial pyruvate carrier; MTF1, metal regulatory transcription factor 1; PDHA1, pyruvate dehydrogenase E1 subunit *α*1; PDHB, pyruvate dehydrogenase E1 subunit *β*; SNV, single nucleotide variation; TCA, tricarboxylic acid; TF, transcription factor.

New cell death mechanisms often lead to the identification of novel tumor therapeutic targets and development of personalized treatment strategies for patients. For example, cells that undergo mitochondrial respiration and those with high levels of lipoylated proteins are highly sensitive to cuproptosis, indicating that Cu ionophore therapy may be very effective for tumors with this metabolic profile [26]. It is conceivable that a great deal of future research will focus on the potential impact of cuproptosis on tumor development and how this unique death mechanism can be exploited to improve the prognosis of patients with cancer and to benefit the larger biomedical community. To provide an initial overview and reference point for future studies, we used big data to comprehensively assess the molecular and clinical characteristics of cuproptosis regulators in 33 tumors from a multiomics perspective. The cell model diagram of cuproptosis and the workflow of this study are shown in Figure 1. Our study showed that cuproptosis is closely related to the prognosis of multiple tumors, activation/inhibition of cancer hallmark pathways, and regulation of the tumor microenvironment. Rational use of cuproptosis exhibits great potential for future cancer therapies.

## RESULTS

### Somatic alteration landscape for cuproptosis regulators

**Figure 2:**
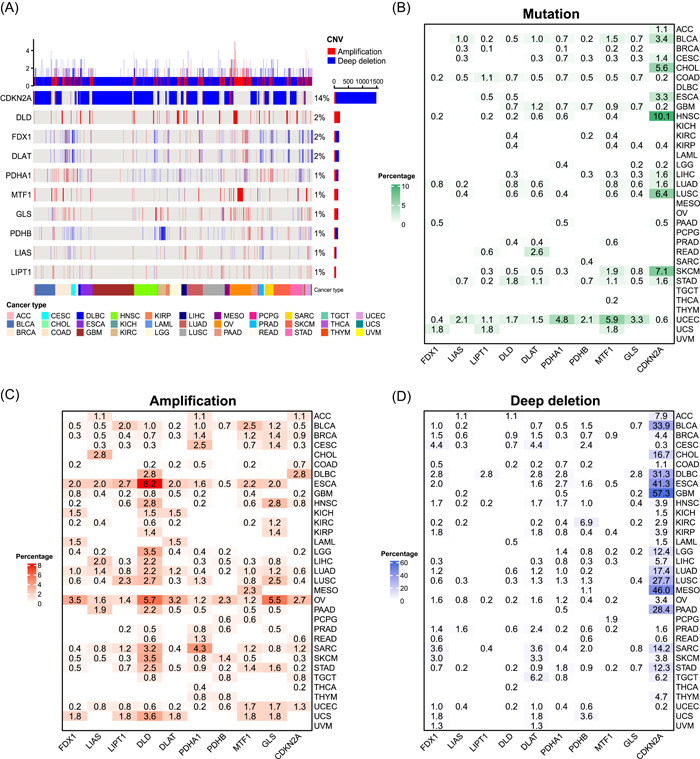
Somatic alterations of cuproptosis regulators in cancer. (A) Landscape of CNV of cuproptosis regulators in cancer. Each row represents a gene, and each column represents a patient. The frequencies of CNV of 10 cuproptosis regulators are presented. Only patients with CNV in the cuproptosis regulators are shown. Alteration rate of each gene is displayed in the right labels. (B–D) Distribution of mutation (B), amplification (C), and deep deletion (D) frequencies over cancer types. The numbers in the graph represent the specific frequency values. The intensity of color is proportional to the frequency. ACC, adrenocortical carcinoma; BLCA, bladder urothelial carcinoma; BRCA, breast invasive carcinoma; CDKN2A, cyclin‐dependent kinase inhibitor 2A; CESC, cervical squamous cell carcinoma and endocervical adenocarcinoma; CHOL, cholangiocarcinoma; CNV, copy number variation; COAD, colon adenocarcinoma; DLAT, dihydrolipoamide *S*‐acetyltransferase; DLBC, diffuse large B‐cell lymphoma; DLD, dihydrolipoamide dehydrogenase; ESCA, esophageal carcinoma; FDX1, ferredoxin 1; GBM, glioblastoma multiforme; GLS, glutaminase; HNSC, head and neck squamous cell carcinoma; KICH, kidney chromophobe; KIRC, kidney renal clear cell carcinoma; KIRP, kidney renal papillary cell carcinoma; LAML, acute myeloid leukemia; LGG, low‐grade glioma; LIAS, lipoyl synthase; LIHC, liver hepatocellular carcinoma; LIPT1, lipolytransferase 1; LUAD, lung adenocarcinoma; LUSC, lung squamous cell carcinoma; MESO, mesothelioma; MTF1, metal regulatory transcription factor 1; OV, ovarian serous cystadenocarcinoma; PAAD, pancreatic adenocarcinoma; PCPG, pheochromocytoma and paraganglioma; PDHA1, pyruvate dehydrogenase E1 subunit *α*1; PDHB, pyruvate dehydrogenase E1 subunit *β*; PRAD, prostate adenocarcinoma; READ, rectum adenocarcinoma; SARC, sarcoma; SKCM, skin cutaneous melanoma; STAD, stomach adenocarcinoma; TGCT, testicular germ cell tumors; THCA, thyroid carcinoma; THYM, thymoma; UCEC, uterine corpus endometrial carcinoma; UCS, uterine carcinosarcoma; UVM, thymoma and uveal melanoma.

To understand the genomic alterations of the 10 cuproptosis regulators in tumors, we analyzed single nucleotide variation (SNV) and copy number variation (CNV) data from 10,680 pan‐cancer samples and portrayed SNV and CNV landscapes. Overall, the CNV frequencies were very low for almost all cuproptosis regulators (1%–2%), except *CDKN2A* that showed CNV frequency in up to 14% of the tumors (Figure 2A). Most CNV in *CDKN2A* were deep deletions. Further, the SNV landscape showed that the SNV frequencies of all cuproptosis regulators were low (0%–2%) and the SNV types of most cuproptosis regulators were dominated by missense mutations (Supporting Information Figure S1). We characterized the somatic alterations of these cuproptosis regulators in each of the 33 tumors to better understand the landscape of different tumor types. Different tumor types had different mutation patterns. All 10 cuproptosis regulators exhibited mutations in colon adenocarcinoma (COAD) and uterine corpus endometrial carcinoma (UCEC), whereas no mutations were observed in diffuse large B‐cell lymphoma (DLBC), kidney chromophobe (KICH), acute myeloid leukemia (LAML), mesothelioma (MESO), ovarian serous cystadenocarcinoma (OV), pheochromocytoma and paraganglioma (PCPG), testicular germ cell tumors (TGCT), and thymoma and uveal melanoma (UVM) (Figure 2B). In addition, the highest mutation frequency of *CNKN2A* was observed in the head and neck squamous cell carcinoma (HNSC) (10.1%). For amplification, all cuproptosis regulators in bladder urothelial carcinoma (BLCA), lung adenocarcinoma (LUAD), OV, and UCEC were amplified, whereas all those in UVM remained unchanged (Figure 2C). *DLD* in esophageal carcinoma (ESCA) had the highest amplification frequency (8.2%). A high percentage of *CDKN2A* deep deletion was observed in many tumor types (Figure 2D). Among them, more than half of the glioblastoma multiforme samples showed a deep deletion of *CDKN2A*, while UVM only showed a low percentage of deletions in *FDX1* (1.3%) and *DLAT* (1.3%). Overall, cuproptosis regulators show a heterogeneous pattern of somatic alterations in different tumor types. Given that gene amplification and deep deletion largely mediate aberrant gene expression [28], we explored the effects of amplification and deep deletion on cuproptosis regulator expression in cancer. Consistent with expectations, amplification samples had the highest gene expression and deep deletion samples had the lowest gene expression among all 10 cuproptosis regulators (Supporting Information Figure S2). This suggests that CNV affects the expression levels of cuproptosis regulators in tumors.

### Gene expression patterns for cuproptosis regulators

To characterize the gene expression patterns of cuproptosis regulators, we first explored the interaction relationships among the regulators using the STRING database. As shown in Supporting Information Figure S3, the seven positive regulators and the negative regulator, *GLS*, formed an interaction network, whereas *MTF1* and *CDKN2A* did not interact with other regulators. We further explored the distribution of regulator expression in different normal tissues. Overall, the expression of the regulators was evenly distributed in different tissues, with the highest expression of *FDX1* in the adrenal gland and very low expression of *CDKN2A* in the bone marrow (Supporting Information Figure S4).

**Figure 3:**
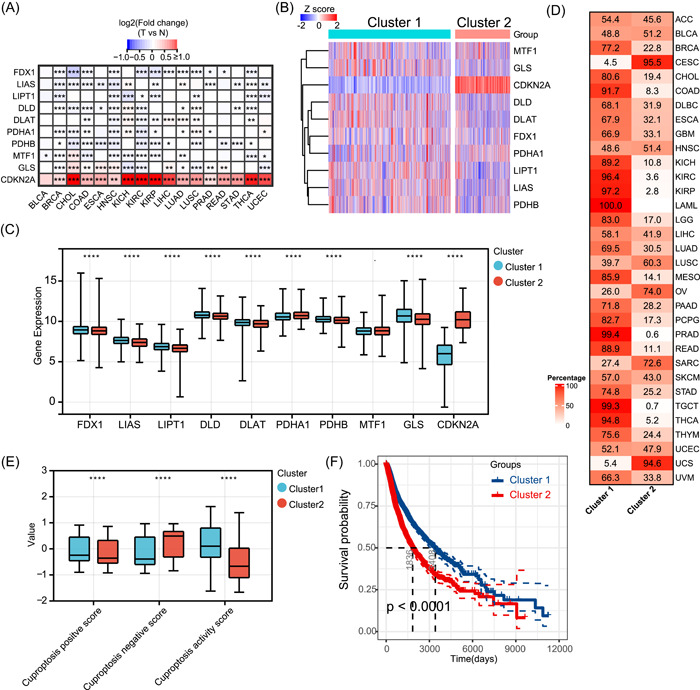
Gene expression patterns of cuproptosis regulators. (A) The mRNA differences between tumor samples and adjacent normal samples. Red indicates high expression in tumor, and blue indicates low expression. *p < 0.05, **p < 0.01, and ***p < 0.001. (B) Unsupervised consensus clustering of cuproptosis regulator expression revealed two distinct clusters. Each row represents a cuproptosis regulator, and each column is a patient. Red indicates high expression, and blue indicates low expression. The expression data were normalized by *z* score. (C) Boxplots showing the differences between 10 cuproptosis regulators in the two clusters. The differences were tested by Student's *t* test. ****p < 0.0001. (D) Sample distribution in the two clusters. Each row represents a cancer type, and each column represents a cluster. The number and red intensity in each box show the percentage of samples classified in the corresponding cluster. (E) Boxplots showing the differences between cuproptosis‐positive score, cuproptosis‐negative score, and cuproptosis activity score in the two clusters. The differences were tested by Student's *t* test. ****p < 0.0001. (F) The Kaplan–Meier curve showing the difference in OS between Clusters 1 and 2. Cluster 1 is indicated in the blue line and Cluster 2 in red. ACC, adrenocortical carcinoma; BLCA, bladder urothelial carcinoma; BRCA, breast invasive carcinoma; CDKN2A, cyclin‐dependent kinase inhibitor 2A; CESC, cervical squamous cell carcinoma and endocervical adenocarcinoma; CHOL, cholangiocarcinoma; CNV, copy number variation; COAD, colon adenocarcinoma; DLAT, dihydrolipoamide *S*‐acetyltransferase; DLBC, diffuse large B‐cell lymphoma; DLD, dihydrolipoamide dehydrogenase; ESCA, esophageal carcinoma; FDX1, ferredoxin 1; GBM, glioblastoma multiforme; GLS, glutaminase; HNSC, head and neck squamous cell carcinoma; KICH, kidney chromophobe; KIRC, kidney renal clear cell carcinoma; KIRP, kidney renal papillary cell carcinoma; LAML, acute myeloid leukemia; LGG, low‐grade glioma; LIAS, lipoyl synthase; LIHC, liver hepatocellular carcinoma; LIPT1, lipolytransferase 1; LUAD, lung adenocarcinoma; LUSC, lung squamous cell carcinoma; MESO, mesothelioma; mRNA, messenger RNA; MTF1, metal regulatory transcription factor 1; OS, overall survival; OV, ovarian serous cystadenocarcinoma; PAAD, pancreatic adenocarcinoma; PCPG, pheochromocytoma and paraganglioma; PDHA1, pyruvate dehydrogenase E1 subunit *α*1; PDHB, pyruvate dehydrogenase E1 subunit *β*; PRAD, prostate adenocarcinoma; READ, rectum adenocarcinoma; SARC, sarcoma; SKCM, skin cutaneous melanoma; STAD, stomach adenocarcinoma; TGCT, testicular germ cell tumors; THCA, thyroid carcinoma; THYM, thymoma; UCEC, uterine corpus endometrial carcinoma; UCS, uterine carcinosarcoma; UVM, thymoma and uveal melanoma.

Differential expression analysis of paired normal and tumor tissues revealed that cuproptosis regulators were aberrantly expressed in 17 tumors (Figure 3A). *FDX1* expression was downregulated in 12 tumors, *LIPT1* expression was downregulated in eight tumors, and *CDKN2A* expression was significantly upregulated in 16 tumors. Other regulators showed heterogeneous expression patterns; for example, *LIAS* expression was downregulated in most tumors, whereas its expression was increased in KICH and LUAD. Subsequently, we explored the role of coexpression of the regulators in 33 tumors. The expression correlations prevalent among all regulators in all tumor types are shown in Supporting Information Table S1. *FDX1* expression was significantly positively correlated with the expression levels of six other positive regulators in most tumors. This suggests that cuproptosis regulators in tumors may coregulate their cuproptosis activity.

To better understand the expression landscape of cuproptosis regulators in tumors, we performed unsupervised consensus clustering based on regulator messenger RNA (mRNA) expression for all samples of the 33 tumor types. On the basis of the consensus cumulative distribution function and delta area, all tumors were distinctly divided into two different sample clusters (Figure 3B). Compared with cluster 2, cluster 1 had higher expression levels of *FDX1*, *LIAS*, *LIPT1*, *DLD*, *DLAT*, *PDHB*, and *GLS*, and significantly lower expression levels of *CDKN2A* (Figure 3C). Examination of the distribution of all cancer types in the two clusters revealed that all LAML, most COAD, three types of kidney cancers (KICH, kidney renal clear cell carcinoma [KIRC], and kidney renal papillary cell carcinoma [KIRP]), prostate adenocarcinoma (PRAD), rectum adenocarcinoma, TGCT, and thyroid carcinoma (THCA) were distributed in cluster 1, and most of the three types of gynecologic tumors (cervical squamous cell carcinoma and endocervical adenocarcinoma [CESC], OV, and uterine carcinosarcoma) were distributed in cluster 2 (Figure 3D). As cuproptosis in tumors is regulated by 10 regulators, the expression of any individual regulator can hardly reflect the overall level of cuproptosis. Therefore, we first proposed the cuproptosis‐positive score, cuproptosis‐negative score, and cuproptosis activity score based on the mRNA expression levels of positive and negative regulators of cuproptosis. In the pan‐cancer context, the positive cuproptosis score was positively correlated with the expression of all positive regulators, and the negative cuproptosis score was positively correlated with the expression of all negative regulators (Supporting Information Figure S5). The cuproptosis activity score was positively correlated with the cuproptosis‐positive score as well as positive regulator expression and negatively correlated with the cuproptosis‐negative score as well as negative regulator expression; thus, the cuproptosis activity score integrated the expression abundance of all regulators and better reflected the overall cuproptosis level. Subsequently, we found that cluster 1 had significantly higher cuproptosis‐positive and activity scores, and lower negative scores than cluster 2 (Figure 3E), suggesting that cluster 1 had a relatively higher level of cuproptosis. Survival analysis showed that cluster 1 had a significantly better overall prognosis than cluster 2 (Figure 3F), implying that the cuproptosis level may influence the survival of patients with different tumor types.

To identify potential cuproptosis phenotype‐related genes, we performed a correlation analysis based on cuproptosis activity. A total of 1079 cuproptosis phenotype‐related genes were identified (Supporting Information Table S2). Through Gene ontology (GO) enrichment analysis and Kyoto encyclopedia of genes and genomes (KEGG) pathway analysis, we found that cuproptosis phenotype‐related genes were mainly involved in energy metabolism‐related biological processes such as aerobic respiration and mitochondrial respiration and pathways, such as oxidative phosphorylation and tricarboxylic acid (TCA) cycle (Supporting Information Figure S6), which is consistent with the study of Tsvetkov et al. [26].

### Methylation analysis of cuproptosis regulators

**Figure 4:**
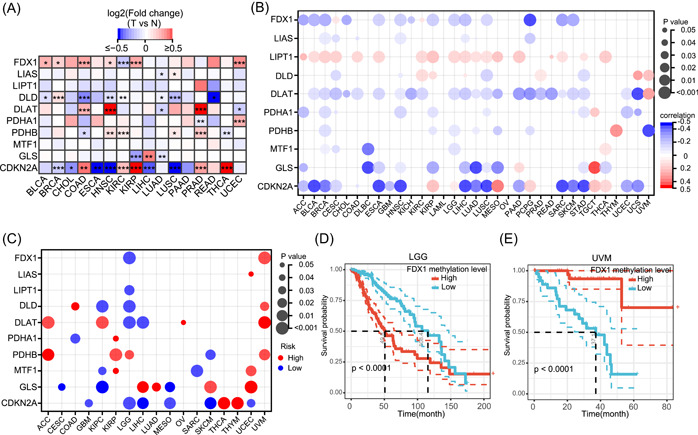
Methylation of cuproptosis regulators. (A) The methylation differences between tumor samples and adjacent normal samples. Red indicates increased methylation in tumors, and blue indicates decreased methylation. *p < 0.05, **p < 0.01, and ***p < 0.001. (B) Correlation between methylation and mRNA gene expression. Blue points represent a negative correlation and red points represent a positive correlation, where the darker the color, the higher the correlation. (C) Relationship between cuproptosis regulators methylation and survival in cancer. Red dots indicate that hypermethylation is associated with worse survival and blue dots indicate that hypermethylation is associated with better survival. The size of the point represents the statistical significance, where the larger the dot size, the higher the statistical significance. Only tumor types with significance are shown. (D, E) Kaplan–Meier curves showing the difference in OS between *FDX1* hypermethylation group and *FDX1* hypomethylation group in LGG (D) and UVM (E). Hypermethylation group is indicated in red line and hypomethylation group in blue. ACC, adrenocortical carcinoma; BLCA, bladder urothelial carcinoma; BRCA, breast invasive carcinoma; CDKN2A, cyclin‐dependent kinase inhibitor 2A; CESC, cervical squamous cell carcinoma and endocervical adenocarcinoma; CHOL, cholangiocarcinoma; CNV, copy number variation; COAD, colon adenocarcinoma; DLAT, dihydrolipoamide *S*‐acetyltransferase; DLBC, diffuse large B‐cell lymphoma; DLD, dihydrolipoamide dehydrogenase; ESCA, esophageal carcinoma; FDX1, ferredoxin 1; GBM, glioblastoma multiforme; GLS, glutaminase; HNSC, head and neck squamous cell carcinoma; KICH, kidney chromophobe; KIRC, kidney renal clear cell carcinoma; KIRP, kidney renal papillary cell carcinoma; LAML, acute myeloid leukemia; LGG, low‐grade glioma; LIAS, lipoyl synthase; LIHC, liver hepatocellular carcinoma; LIPT1, lipolytransferase 1; LUAD, lung adenocarcinoma; LUSC, lung squamous cell carcinoma; MESO, mesothelioma; MTF1, metal regulatory transcription factor 1; OS, overall survival; OV, ovarian serous cystadenocarcinoma; PAAD, pancreatic adenocarcinoma; PCPG, pheochromocytoma and paraganglioma; PDHA1, pyruvate dehydrogenase E1 subunit *α*1; PDHB, pyruvate dehydrogenase E1 subunit *β*; PRAD, prostate adenocarcinoma; READ, rectum adenocarcinoma; SARC, sarcoma; SKCM, skin cutaneous melanoma; STAD, stomach adenocarcinoma; TGCT, testicular germ cell tumors; THCA, thyroid carcinoma; THYM, thymoma; UCEC, uterine corpus endometrial carcinoma; UCS, uterine carcinosarcoma; UVM, thymoma and uveal melanoma.

Methylation in the promoter regions of genes largely regulates the gene expression, and hypermethylation generally suppresses the gene expression [29]. However, there are some special cases where hypermethylation of the promoter region may enhance the expression of a gene, such as *hTERT* [30]*.* To explore the methylation alterations in cuproptosis regulators, we first compared the methylation differences in the regulators between paired normal and tumor tissues. As shown in Figure 4A, the methylation levels of regulators varied across all 16 tumors. Methylation alterations in regulators were heterogeneous in different tumor types. For example, the methylation levels of *FDX1* were elevated in BLCA, breast invasive carcinoma (BRCA), COAD, HNSC, KIRP, and UCEC tumor tissues compared with paired normal tissues, while they were decreased in KIRC. Notably, *MTF1* did not show altered methylation levels in any of the tumor types. Furthermore, we analyzed the correlation between regulatory methylation levels and mRNA expression levels in 33 tumor types. In most tumor types, the promoter methylation levels of *FDX1*, *DLAT*, *GLS*, and *CDKN2A* were negatively correlated with the mRNA expression levels (Figure 4B). The methylation levels of *LIAS*, *DLD*, *PDHA1*, *PDHB*, and *MTF1* were negatively or positively correlated with the mRNA expression levels in specific tumor types. For example, *MTF1* was negatively correlated with BRCA, CESC, DLBC, and brain low‐grade glioma (LGG) and positively correlated with THCA (Figure 4B). Notably, the methylation levels of *LIPT1* were positively correlated to the mRNA expression levels in most tumor types. Survival analysis revealed that the methylation levels of cuproptosis regulators were correlated with the overall survival (OS) in 17 tumor types and that the correlations were tumor type‐dependent (Figure 4C). For instance, *FDX1* hypermethylation was associated with poor OS in LGG and good OS in UVM (Figure 4D,E).

### MicroRNAs (miRNAs), long noncoding RNAs (lncRNAs), and transcription factors (TFs) regulate the expression levels of cuproptosis regulators

**Figure 5:**
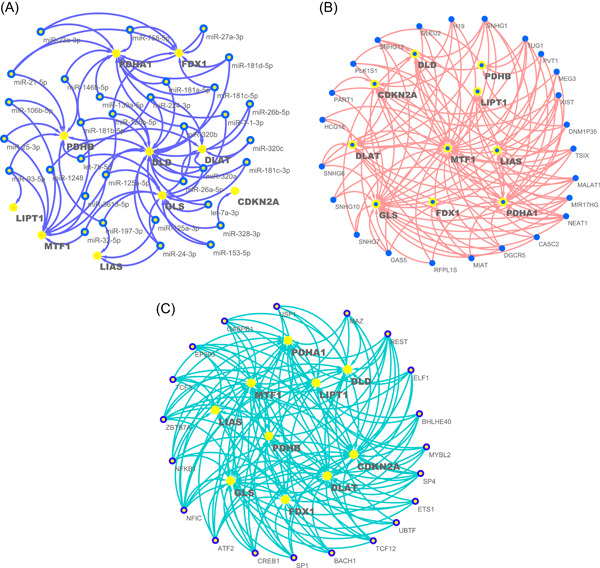
miRNA, lncRNA, and TF regulatory networks. (A) The miRNA–mRNA regulatory network showing miRNAs target at least two cuproptosis regulators. (B) The lncRNA–mRNA regulatory network showing lncRNAs target at least three cuproptosis regulators. (C) The TF–mRNA regulatory network showing TFs target at least five cuproptosis regulators. CDKN2A, cyclin‐dependent kinase inhibitor 2A; DLAT, dihydrolipoamide *S*‐acetyltransferase; DLD, dihydrolipoamide dehydrogenase; DX1, ferredoxin 1; GLS, glutaminase; LIAS, lipoyl synthase; LIPT1, lipolytransferase 1; lncRNA, long noncoding RNA; miRNA, microRNA; mRNA, messenger RNA; MTF1, metal regulatory transcription factor 1; PDHA1, pyruvate dehydrogenase E1 subunit *α*1; PDHB, pyruvate dehydrogenase E1 subunit *β*; TF, transcription factor.

In previous results of this study, we described the regulation of cuproptosis regulators expression by CNV and DNA methylation. miRNAs are gene expression repressors that can regulate gene expression posttranscriptionally by binding to the 3′‐untranslated regions of target mRNAs [31, 32]. To comprehensively explore miRNAs that may regulate cuproptosis regulators, we screened all miRNAs that could target the 3′‐untranslated regions of these regulators. In Supporting Information Table S3, we have listed all potential miRNA–mRNA pairs that may target different cuproptosis regulators in each tumor type after threshold screening as a reference for future cuproptosis‐related miRNA studies. Notably, 174 miRNA–mRNA pairs, including 127 miRNAs, were present in at least five tumor types. Interestingly, 33 of the 127 miRNAs targeted at least two cuproptosis regulators, constituting a miRNA regulatory network (Figure 5A). Given that these miRNAs target multiple regulators in multiple tumors, these miRNAs may be potential miRNAs regulating cuproptosis.

LncRNAs are important regulators of gene expression and play important roles in transcription, translation, and posttranslational modifications [33]. Therefore, we combined the pan‐cancer analysis of lncRNA regulation with gene expression correlation analysis data to filter potential lncRNA–mRNA pairs regulating cuproptosis in different tumor types (Supporting Information Table S4) [34]. A total of 168 lncRNA–mRNA pairs were identified involving 72 lncRNAs, 24 of which targeted at least three cuproptosis regulators and constituted a lncRNA regulatory network (Figure 5B).

TFs are key regulators of gene transcription and expression, and dysregulated TFs mediate aberrant gene expression and represent a unique class of drug targets [35]. We examined a series of TFs listed in a previous pan‐cancer study and identified a total of 429 TF–mRNA pairs containing 196 different TFs [34], 20 of which regulated at least five cuproptosis regulators (Figure 5C). Importantly, nuclear factor I C (*NFIC*) targeted nine cuproptosis regulators, suggesting that it may be a key TF mediating cuproptosis in tumors. Supporting Information Table S5 lists all TF–mRNA pairs that may target cuproptosis regulators in different tumor types.

### Cuproptosis activity predicts the prognosis of patients with cancer

**Figure 6:**
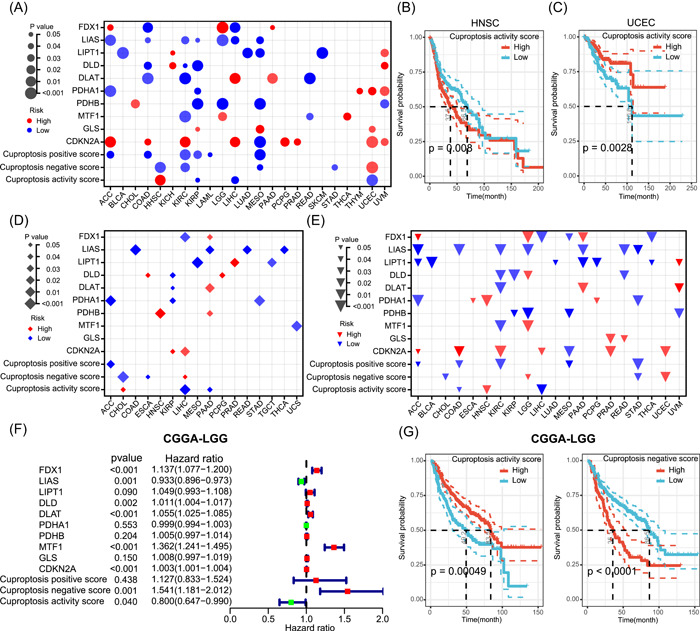
Prognostic value of cuproptosis. OS (A), DFI (D), and PFI (E) analyses of cuproptosis regulators and cuproptosis scores. The dot size represents the significance of the effects of cuproptosis regulators or scores on survival in each cancer type, the *p* value was obtained from a log‐rank test. Only tumor types with significance are shown. Red dots indicate that high gene expression or high score is associated with worse survival and blue dots indicate that the high gene expression or high score is associated with better survival. (B, C) Kaplan–Meier curves showing the difference in OS between high‐cuproptosis activity score group and low cuproptosis activity score group in HNSC (B) and UCEC (C). High‐cuproptosis activity score group is indicated in the red line and the low cuproptosis activity score group in blue. (F) OS analysis of cuproptosis regulators and cuproptosis scores in CGGA‐LGG cohort. *p* Values and hazard ratios were calculated by Cox regression analysis. Red dots indicate that high gene expression or high score is associated with worse OS and green dots indicate that the high gene expression or high score is associated with better OS. (G) Kaplan–Meier curves showing the difference in OS between high‐cuproptosis activity/negative score group and low cuproptosis activity/negative score group in CGGA‐LGG cohort. ACC, adrenocortical carcinoma; BLCA, bladder urothelial carcinoma; BRCA, breast invasive carcinoma; CDKN2A, cyclin‐dependent kinase inhibitor 2A; CESC, cervical squamous cell carcinoma and endocervical adenocarcinoma; CGGA, Chinese Glioma Genome Atlas; CHOL, cholangiocarcinoma; CNV, copy number variation; COAD, colon adenocarcinoma; DFI, disease‐free interval; DLAT, dihydrolipoamide *S*‐acetyltransferase; DLBC, diffuse large B‐cell lymphoma; DLD, dihydrolipoamide dehydrogenase; ESCA, esophageal carcinoma; FDX1, ferredoxin 1; GBM, glioblastoma multiforme; GLS, glutaminase; HNSC, head and neck squamous cell carcinoma; KICH, kidney chromophobe; KIRC, kidney renal clear cell carcinoma; KIRP, kidney renal papillary cell carcinoma; LAML, acute myeloid leukemia; LGG, low‐grade glioma; LIAS, lipoyl synthase; LIHC, liver hepatocellular carcinoma; LIPT1, lipolytransferase 1; LUAD, lung adenocarcinoma; LUSC, lung squamous cell carcinoma; MESO, mesothelioma; MTF1, metal regulatory transcription factor 1; OS, overall survival; PAAD, pancreatic adenocarcinoma; PCPG, pheochromocytoma and paraganglioma; PDHA1, pyruvate dehydrogenase E1 subunit *α*1; PDHB, pyruvate dehydrogenase E1 subunit *β*; PFI, progression‐free interval; PRAD, prostate adenocarcinoma; READ, rectum adenocarcinoma; SARC, sarcoma; SKCM, skin cutaneous melanoma; STAD, stomach adenocarcinoma; TCGA, The Cancer Genome Atlas; TGCT, testicular germ cell tumors; THCA, thyroid carcinoma; THYM, thymoma; UCEC, uterine corpus endometrial carcinoma; UCS, uterine carcinosarcoma; UVM, thymoma and uveal melanoma.

The results of this study demonstrated that cuproptosis activity predicted the OS in a pan‐cancer context. To further clarify the impact of cuproptosis on patient survival, we explored the prognostic predictive roles of cuproptosis regulators and cuproptosis scores for different tumor types. We performed Cox regression analysis on cuproptosis regulators or scores to calculate the survival risk and used the log‐rank test to determine significance after dividing different tumors into two groups according to the median of cuproptosis regulators or scores. As shown in Figure 6A, cuproptosis regulators and scores had different prognostic roles in different tumor types. High *LIAS* expression was associated with better OS in adrenocortical carcinoma (ACC), COAD, KIRC, KIRP, LGG, liver hepatocellular carcinoma (LIHC), and MESO, while high *CDKN2A* expression was associated with poor OS in ACC, COAD, KIRC, LIHC, PCPG, PRAD, UCEC, and UVM. In addition, an elevated cuproptosis activity score was associated with poorer OS in HNSC and better OS in patients with KIRP, LIHC, and UCEC. Figure 6B,C shows the Kaplan–Meier survival curves between the high and low cuproptosis activity score groups after dividing the samples into two groups according to the median value in HNSC and UCEC, respectively. To further confirm the effect of cuproptosis on the OS of patients, we performed Kaplan–Meier survival curve analysis after dividing the patients into two groups according to the best cutoff value of the cuproptosis activity score. The results showed that the cuproptosis activity was significantly associated with the OS in 13 of the 33 tumor types (Supporting Information Figure S7). Among them, higher cuproptosis activity predicted better OS in COAD, DLBC, KIRP, LAML, LGG, LIHC, lung squamous cell carcinoma (LUSC), sarcoma, and UCEC and also predicted poorer OS in HNSC, LUAD, skin cutaneous melanoma, and UVM. In addition to OS, we also tested the relationship between cuproptosis and disease‐free interval (DFI) and progression‐free interval (PFI) in patients with tumors. We found that the cuproptosis regulator levels and activity scores were strongly correlated with DFI in 16 tumor types and PFI in 20 tumor types, with the correlation depending on the tumor type (Figure 6D,E). Further, we performed external validation using the LGG cohort from the Chinese Glioma Genome Atlas (CGGA). As shown in Figure 6F, Cox regression analysis showed that *FDX1*, *MTF1*, and cuproptosis‐negative scores were associated with poorer OS in LGG, while *LIAS* and cuproptosis activity scores were associated with better OS. Kaplan–Meier curves further verified the prognosis value of cuproptosis scores in LGG (Figure 6G), which is consistent with the results in The Cancer Genome Atlas (TCGA) database. These results suggest that cuproptosis is correlated with the prognosis of specific tumor types and can predict patient survival.

### Pathway activity analyses of cuproptosis

**Figure 7:**
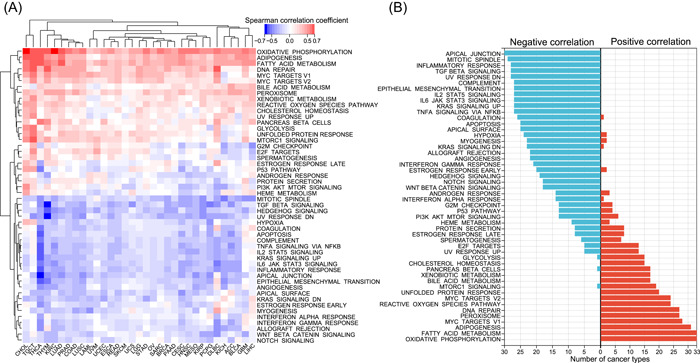
Cuproptosis correlated hallmark pathways in cancer. (A) The heatmap showing the correlation between cuproptosis activity and hallmark pathway activity in each cancer type. Each column is a cancer type, and each row is a hallmark set. Red represents a positive correlation, blue represents a negative correlation. Unsupervised clustering used Euclidean distance metrics with complete linkage. (B) Bar plots showing a number of cancer types having negative (blue) and positive (red) correlations between cuproptosis activity and hallmark pathways. ACC, adrenocortical carcinoma; BLCA, bladder urothelial carcinoma; BRCA, breast invasive carcinoma; CESC, cervical squamous cell carcinoma and endocervical adenocarcinoma; CHOL, cholangiocarcinoma; COAD, colon adenocarcinoma; DLBC, diffuse large B‐cell lymphoma; ESCA, esophageal carcinoma; GBM, glioblastoma multiforme; HNSC, head and neck squamous cell carcinoma; KIRC, kidney renal clear cell carcinoma; KIRC, kidney renal clear cell carcinoma; KIRP, kidney renal papillary cell carcinoma; LAML, acute myeloid leukemia; LGG, low‐grade glioma; LIHC, liver hepatocellular carcinoma; LUAD, lung adenocarcinoma; LUSC, lung squamous cell carcinoma; MESO, mesothelioma; OV, ovarian serous cystadenocarcinoma; PAAD, pancreatic adenocarcinoma; PCPG, pheochromocytoma and paraganglioma; PRAD, prostate adenocarcinoma; READ, rectum adenocarcinoma; SARC, sarcoma; SKCM, skin cutaneous melanoma; STAD, stomach adenocarcinoma; TGCT, testicular germ cell tumors; THCA, thyroid carcinoma; THYM, thymoma; UCEC, uterine corpus endometrial carcinoma; UCS, uterine carcinosarcoma; UVM, thymoma and uveal melanoma.

Our results demonstrated the dysregulation and prognostic role of cuproptosis in tumors; however, the specific tumor‐related pathways involved in cuproptosis remain unknown. Therefore, we first inferred the enrichment level of 50 cancer hallmark gene sets in all tumor samples from 33 tumor types (Supporting Information Table S6), which comprehensively reflected the biological processes associated with tumors [36]. Subsequently, we calculated the correlation of the cuproptosis activity score with all hallmark gene sets in each tumor type and generated a heatmap (Figure 7A; Supporting Information Table S7). Cuproptosis activity was positively correlated with approximately half of the hallmark gene sets in most tumor types and negatively correlated with the other half, indicating that cuproptosis plays a key role in tumors (Figure 7A). Notably, cuproptosis activity was significantly positively correlated with oxidative phosphorylation in all 33 tumor types (Figure 7B), which is consistent with the study by Tsvetkov et al. that cuproptosis is dependent on mitochondrial respiration and TCA cycle [26], further suggesting that the cuproptosis activity score can reliably reflect the cuproptosis level. In addition, cuproptosis was also significantly negatively correlated with hypoxia in 24 tumor types (Figure 7B), and Tsvetkov et al. found that hypoxia (1% O_2_) reduces cuproptosis sensitivity by obliging cells to rely on glycolysis rather than oxidative phosphorylation [26, 27]. Cuproptosis was also significantly positively correlated to fatty acid metabolism in all tumor types (Figure 7B). Additionally, cuproptosis was negatively associated with the apical junction, mitotic spindle, epithelial‐mesenchymal transition (EMT), transforming growth factor *β* (TGF‐*β)*, Kirsten RAt Sarcoma, and tumor necrosis factor‐*α* signaling pathways in more than 25 cancer types, confirming the important regulatory role of cuproptosis in tumor metastasis and growth. Strikingly, cuproptosis was negatively associated with immune‐related pathways, including the inflammatory response, complement, interleukin‐6/Janus kinase/signal transducer and activator of transcription 3 signaling, and interferon gamma response pathways, in most tumor types (Figure 7B). Given that cuproptosis was positively associated with DNA repair in 27 tumor types, we further explored whether cuproptosis was related to genomic instability. Consistent with expectations, cuproptosis was negatively associated with indicators related to homologous recombination deficiency (HRD) in 11 tumor types, but negatively associated with mutational burden in only three tumor types (Supporting Information Figure S8). In addition, since *CDKN2A* is a well‐known cell senescence and cell cycle marker, we further explored the relationship of cuproptosis with other cell senescence and cycle markers. As shown in Supporting Information Figure S9A, in almost all tumor types, cuporotosis was negatively associated with most cell senescence markers. For cell cycle markers, 15 markers including *CDK7* were positively correlated with cuporotosis in almost all tumor types, whereas most other cell cycle markers were predominantly negatively correlated with cuporotosis in tumors (Supporting Information Figure S9B). Of the seven tumor types, including UVM, cuporotosis was positively correlated with most cell cycle markers. In summary, the above results suggest that cuproptosis is involved in numerous biological processes in tumors.

### Cuproptosis‐associated immune characteristics

**Figure 8:**
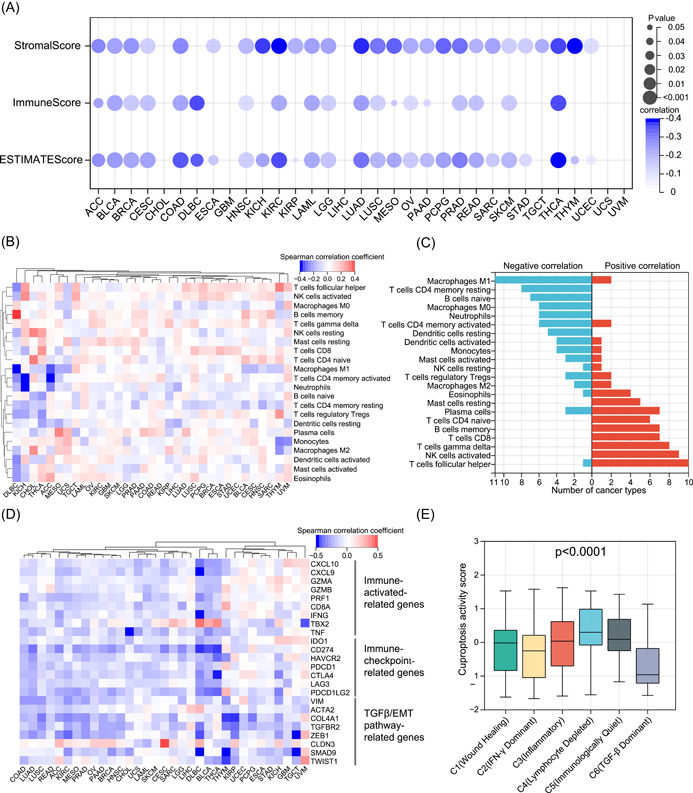
Cuproptosis correlates with tumor immune characteristics. (A) Correlation between cuproptosis activity and ImmuneScore, StromalScore, and ESTIMATEScore. Blue points represent a negative correlation, where the darker the color, the higher the correlation. (B) The heatmap showing the correlation between cuproptosis activity and the abundance of 22 immune cells in each cancer type. Each column is a cancer type, and each row is an immune cell. Red represents a positive correlation, blue represents a negative correlation. Unsupervised clustering used Euclidean distance metrics with complete linkage. (C) Bar plots showing a number of cancer types having negative (blue) and positive (red) correlations between cuproptosis activity and 22 immune cells. (D) The heatmap showing the correlation between cuproptosis activity and immunomodulator expression in each cancer type. Each column is a cancer type, and each row is an immunomodulator. Red represents a positive correlation, blue represents a negative correlation. Unsupervised clustering used Euclidean distance metrics with complete linkage. (E) Boxplots represent the differences in cuproptosis activity between immune subtypes (C1–C6). Student's *t* test was used for the comparison between two immune subtypes. All comparison *p* < 0.0001. ACC, adrenocortical carcinoma; BLCA, bladder urothelial carcinoma; BRCA, breast invasive carcinoma; CESC, cervical squamous cell carcinoma and endocervical adenocarcinoma; CHOL, cholangiocarcinoma; COAD, colon adenocarcinoma; DLBC, diffuse large B‐cell lymphoma; EMT, epithelial‐mesenchymal transition; ESCA, esophageal carcinoma; GBM, glioblastoma multiforme; HNSC, head and neck squamous cell carcinoma; KIRC, kidney renal clear cell carcinoma; KIRC, kidney renal clear cell carcinoma; KIRP, kidney renal papillary cell carcinoma; LAML, acute myeloid leukemia; LGG, low‐grade glioma; LIHC, liver hepatocellular carcinoma; LUAD, lung adenocarcinoma; LUSC, lung squamous cell carcinoma; MESO, mesothelioma; OV, ovarian serous cystadenocarcinoma; PAAD, pancreatic adenocarcinoma; PCPG, pheochromocytoma and paraganglioma; PRAD, prostate adenocarcinoma; READ, rectum adenocarcinoma; SARC, sarcoma; SKCM, skin cutaneous melanoma; STAD, stomach adenocarcinoma; TGCT, testicular germ cell tumors; TGF*β*, transforming growth factor *β*; THCA, thyroid carcinoma; THYM, thymoma; UCEC, uterine corpus endometrial carcinoma; UCS, uterine carcinosarcoma; UVM, thymoma and uveal melanoma.

In pathway activity analyses, we observed significant correlations between cuproptosis and immune‐related pathways. To better reveal the intrinsic link between cuproptosis and tumor immunity, we first inferred the overall immune (ImmuneScore) and stromal (StromalScore) infiltration levels in all tumor samples (Supporting Information Table S8). Consistent with the results of the pathway activity analyses, cuproptosis was significantly negatively correlated with the overall immune and stromal cell infiltration levels in most tumor types (Figure 8A). In addition, cuproptosis was negatively correlated with the ESTIMATEScore of tumors, indicating that cuproptosis is positively correlated with tumor purity. To further understand the correlation between cuproptosis and the abundance of different types of immune cells, we collected the infiltration levels of 22 immune cells of all tumor samples from a previous pan‐cancer analysis [37]. We calculated the correlation between cuproptosis and the levels of immune cell infiltration in each tumor type (Supporting Information Table S9). As shown in Figure 8B, there was a heterogeneous correlation pattern between cuproptosis and tumor‐infiltrating immune cells. Of the 11 tumor types, cuproptosis was negatively correlated with the abundance of M1 macrophages, whereas in 10 tumor types, cuproptosis was positively correlated with the abundance of follicular helper T cells (Figure 8C). Overall, cuproptosis was correlated with the abundance of numerous immune cells, and the correlation may vary depending on the tumor type. Immunomodulators are crucial for the response to immunotherapy, and we collected some classical immune activation‐related genes, immune checkpoint‐related genes, and TGF‐*β*/EMT pathway‐related genes from a previous study by Zeng et al. [38]. We then calculated the correlation between cuproptosis and these immunomodulators (Supporting Information Table S10). In general, cuproptosis was negatively correlated with most immunomodulators in most tumor types, which is consistent with the results of previous studies (Figure 8D). Although immune activation‐related genes appear to be positively associated with cuproptosis in some specific tumor types, such as glioblastoma multiforme, this correlation was not significant. Thorsson et al. classified all samples from 33 tumor types into six immune subtypes (C1–C6) with different immune characteristics in a pan‐cancer context and were widely recognized [37]. Therefore, we compared the differences in cuproptosis between the six immune subtypes. As shown in Figure 8E, C4 (lymphocyte depleted) tumors had the highest level of cuproptosis activity, whereas C6 (TGF‐*β*‐dominant) tumors had the lowest cuproptosis activity. This further validated the negative correlation between cuproptosis and the TGF‐*β* pathway. On the basis of this result, we can hypothesize that the lower immune response in high‐cuproptosis tumors may be related to lymphocyte depletion. These results imply a broad link between cuproptosis and tumor immunity.

### Association of cuproptosis with drug sensitivity and immunotherapy outcome

**Figure 9:**
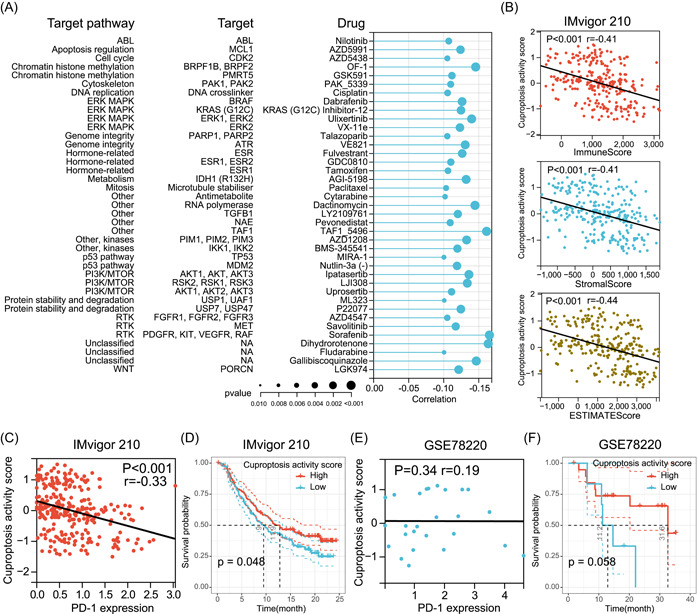
Relationship between cuproptosis activity, drug sensitivity, and immunotherapy outcomes. (A) The correlation between cuproptosis activity and drug sensitivity (IC_50_ value). Each row represents a drug and drug target. The length of the line represents the correlation coefficient. The blue represents a negative correlation, that is, high‐cuproptosis activity correlates with higher drug sensitivity. The size of the point represents the statistical significance, where the larger the dot size, the higher the statistical significance. (B) Correlation of cuproptosis activity with ImmuneScore, StromalScore, and ESTIMATEScore in the IMvigor210 cohort. (C) Correlation of cuproptosis activity with PD‐L1 expression in the IMvigor210 cohort. (D) Kaplan–Meier curve depicts the OS difference between high and low cuproptosis activity groups after anti‐PD‐L1 immunotherapy in the IMvigor210 cohort. (E) Correlation of cuproptosis activity with *PD‐1* expression in the GSE78220 cohort. (F) Kaplan–Meier curve depicts the OS difference between high and low cuproptosis activity groups after anti‐PD‐1 immunotherapy in the GSE78220 cohort. Statistical significance was assessed by a log‐rank test. anti‐PD‐L1, antiprogrammed death ligand‐1; IC_50_, half maximal inhibitory concentration; OS, overall survival; PD‐1, programmed death‐1.

After establishing the association of cuproptosis with numerous cancer hallmark pathways and immune‐related characteristics, we aimed to understand whether cuproptosis could influence patient response to chemotherapy, targeted therapies, and immunotherapy. We integrated gene expression data and drug sensitivity data of cancer cell lines from the Genomics of Drug Sensitivity in Cancer (GDSC) database and analyzed the correlation of cuproptosis with the half maximal inhibitory concentration (IC_50_) of 198 drugs. We observed significant negative correlations between cuproptosis and the IC_50_ of 39 drugs but failed to observe a positive correlation between cuproptosis and the IC_50_ of any drug (Figure 9A). By examining the mechanism of action of these 39 drugs, it was found that some of the targeted pathways had been shown to be negatively associated with cuproptosis activity in the previous results. For example, cuproptosis in the pathway activity analyses was negatively correlated with P53 and PI3K/MTOR pathways in 13 tumor types, and cuproptosis was associated with increased sensitivity to two P53 pathway inhibitors (MIRA‐1 and Nutlin‐3a (−)) and three PI3K/MTOR pathway inhibitors (Ipatasertib, LJI308, and Uprosertib) (Figure 9A), which further enhanced the reliability of our study. In addition, we estimated the drug sensitivity of individual tumor samples in ACC and explored the relationship between IC_50_ and cuproptosis for these samples. Consistent with the previous results, in ACC, cuproptosis was associated with increased sensitivity to four PI3K/MTOR pathway inhibitors (Afuresertib, Ipatasertib, OSI‐027, and Uprosertib) and one P53 pathway inhibitors (Nutlin‐3a (−)) (Supporting Information Table S11). Interestingly, we also found that cuproptosis was associated with increased resistance to 29 drugs in ACC, involving multiple targets such as DNA replication.

Immune checkpoint inhibitor (ICI) therapy is currently the most successful and most common immunotherapy approach [39, 40]. To further investigate whether cuproptosis affects ICI therapy outcomes in tumor patients, we collected a metastatic urothelial cancer cohort receiving antiprogrammed death ligand‐1 (PD‐L1) therapy (IMvigor210) and a metastatic melanoma cohort receiving antiprogrammed death‐1 (PD‐1) therapy (GSE78220) from previous studies [41, 42]. In the IMvigor210 cohort, cuproptosis was found to be negatively correlated with the tumor ImmuneScore, StromalScore, and ESIMATEScore (Figure 9B). More importantly, cuproptosis was negatively correlated with *PD‐L1* expression in this cohort (Figure 9C). Interestingly, Kaplan–Meier survival curve analysis showed that patients with high‐cuproptosis activity had longer survival after anti‐PD‐L1 therapy than those with low cuproptosis activity (Figure 9D). In the GSE78220 cohort, although cuproptosis did not correlate with *PD‐1* expression (Figure 9E), Kaplan–Meier survival curve analysis demonstrated that patients with high‐cuproptosis activity had a better OS after anti‐PD‐1 treatment than those with low cuproptosis activity (Figure 9F). These results provide preliminary evidence of the predictive role of cuproptosis in ICI immunotherapy.

## DISCUSSION

Cu acts as a double‐edged sword, and imbalances in its homeostasis can seriously endanger the human life [43, 44, 45]. Both Cu chelators and Cu ionophores act as promising anticancer agents [17, 19, 20, 46, 47, 48]. Previous studies have suggested that the mechanism of Cu overload toxicity depends on oxidative stress [1, 27]. However, Tsvetkov et al. first revealed cuproptosis, a completely new form of cell death different from the previously known regulated cell death and screened 10 regulators of cuproptosis [26]. Unlike oxidative stress‐dependent regulated cell death processes, such as apoptosis and ferroptosis, cuproptosis is dependent on mitochondrial stress and is induced by Cu binding to lipoylated components of the TCA cycle [26]. As a new and completely different form of regulated cell death that has never been characterized before, cuproptosis‐based tumor research is expected to flourish. Therefore, preliminary studies on the role of cuproptosis in tumors are necessary to further understand the mechanisms of tumor development and explore new clinical therapies. This study provides a comprehensive and integrated characterization of cuproptosis by mining and analyzing multiomics data from more than 10,000 samples of 33 tumor types from TCGA.

Genetic variation analysis revealed that most cuproptosis regulators had a low proportion of somatic alterations in tumors; however, *CDKN2A* underwent extensive somatic alterations in a variety of tumors. This is consistent with the currently available knowledge that *CDKN2A* is involved in cell growth and cycle regulation [49, 50], and that its mutation and loss are important events in many tumors and contribute to multiple tumorigenesis [51, 52]. However, we found that the mRNA expression of *CDKN2A*, a tumor suppressor, was significantly upregulated in 16 tumor types. Although deletion of *CDKN2A* could reduce its expression, *CDKN2A* showed hypomethylation in BRCA, ESCA, HNSC, LIHC, and LUSC, suggesting that *CDKN2A* upregulation in these tumors may be mainly regulated by hypomethylation. Interestingly, *CDKN2A* expression and methylation levels were elevated in both KIRP and THCA, yet *CDKN2A* hypermethylation in both tumors upregulated gene expression, suggesting that high *CDKN2A* expression in KIRP and THCA is also regulated by hypermethylation. This anomaly suggests tumor type‐dependent regulation of gene expression by methylation of *CDKN2A* and warrants further investigation. For some specific tumors, such as LUSC, the expression of *CDKN2A* was significantly elevated despite the presence of substantial deep deletion and hypermethylation, indicating that *CDKN2A* expression was mainly influenced by other mechanisms in these tumors, such as miRNAs, lncRNAs, and TFs. Furthermore, although the oncogenic effects of mutations and loss of *CDKN2A* are well established [53], unexpectedly high *CDKN2A* expression indicates a poor prognosis in a variety of tumors. Similarly, previous studies have shown that high *CDKN2A* expression is associated with poor prognosis in LIHC, COAD, and BLCA [54, 55, 56, 57]. However, the mechanism by which *CDKN2A* acts as a tumor suppressor but leads to poor prognosis has not been clarified. Shi et al. speculated that this might be related to the involvement of *CDKN2A* in the EMT process [56]. The role of *CDKN2A* as a negative regulator of cuproptosis has never been characterized before. Considering that high‐cuproptosis activity was revealed to be a favorable prognostic factor in multiple tumors (including COAD and LIHC) in the present study, it is reasonable to assume that *CDKN2A* may contribute to the poor prognosis of these tumors by negatively regulating cuproptosis activity, which is worthy of in‐depth study in the future to develop suitable targeted agents. In addition, the expression of *CDKN2A* is negatively related to the expression of cuproptosis‐positive regulators (including *FDX1* and *LIPT1*) in multiple tumor types. We believe that the expression of *CDKN2A* may also be affected by other cuproptosis regulators. Therefore, simultaneous targeting of multiple regulators, including *CDKN2A*, may be able to produce synergistic effects, which requires extensive basic and clinical research to verify.

High expression of *FDX1*, the most important positive regulator of cuproptosis, was only associated with a favorable prognosis in COAD and LIHC, which may be due to the upregulation of cuproptosis activity by *FDX1*. However, *FDX1* did not correlate with the prognosis of most tumor types, consistent with the study by Zhang et al., who also showed that *FDX1* was unable to affect proliferation and apoptosis of LUAD cells [58]. Considering the prognostic impact of cuproptosis activity on many tumor types, it is necessary to consider all cuproptosis regulators as a whole. Accordingly, we developed the cuproptosis activity score, and correlation and pathway activity analyses adequately demonstrated that cuproptosis activity scores can reflect the overall cuproptosis level, which provides a reference method for future cuproptosis studies.

The regulatory roles of miRNAs, lncRNAs, and TFs in gene expression are well known [56, 58, 59]; There is no doubt that there must be miRNAs, lncRNAs and TFs that can indirectly regulate cuproptosis. To pave the way for further related studies, we identified potential cuproptosis‐related miRNAs, lncRNAs, and TFs, and constructed regulatory networks. Among the identified miRNAs, some have been verified in previous studies, such as *miR‐10b* targeting *CDKN2A* in gliomas [59], which further supports the reliability of our study. Interestingly and importantly, there were some interactions between the potential miRNA–mRNA pairs and lncRNA‐mRNA pairs that we identified. For example, both lncRNA *MEG3* and *miR‐204* in this study could target *CDKN2A*, and in a previous study *MEG3* was found to regulate inflammation and apoptosis in macrophages via the MEG3/miR‐204/CDKN2A regulatory axis [60]. This suggests that the lncRNAs identified in this study may also regulate cuproptosis regulator expression via miRNAs in the mechanism of competing endogenous RNA [61]. Therefore, it seems feasible and promising to combine the lncRNAs and miRNAs we identified for further study. It is important to mention that the TF *NFIC* targets nine cuproptosis regulators in the regulatory network, implying that *NFIC* may be a key upstream TF of cuproptosis. The role of *NFIC* in the regulation of growth and metastasis of a variety of tumors has been confirmed, and its mechanism involves the EMT process and nuclear factor kappa B pathway [62, 63, 64, 65, 66, 67]. However, the association of *NFIC* with cuproptosis activity has never been characterized; therefore, we hypothesized that *NFIC* could also affect tumor development by regulating cuproptosis activity, which deserves further exploration.

In the pathway activity analyses, we noted strong correlations among cuproptosis, oxidative phosphorylation, and hypoxia, which stem from the mechanism of cuproptosis occurrence that has been elucidated in detail by Tsvetkov et al. [26]. Moreover, we revealed a negative correlation between cuproptosis and multiple tumor‐related pathways, which directly provides a theoretical basis for our findings in the drug sensitivity analysis. In our results, high‐cuproptosis activity was associated with high sensitivity to multiple drugs, implying that Cu ionophores have the potential to synergize with multiple drugs, such as antitumor agents. This finding has been validated in previous experiments. For example, drug sensitivity analysis showed that cuproptosis increased the sensitivity of three PI3K inhibitors, while in the study by Zhang et al., the combination of the Cu ionophore disulfiram with PI3K inhibitors significantly inhibited breast cancer growth in both in vivo and in vitro experiments [68]. In addition to its relevance to tumor‐related pathways, another finding of interest was that cuproptosis was positively correlated with fatty acid metabolism in all tumor types. Tsvetkov et al. only reported that cuproptosis was more sensitive in cells dependent on mitochondrial respiration (oxidative phosphorylation) but did not mention fatty acid metabolism. Fatty acid metabolism is an important component of cancer cell metabolism. In fatty acid metabolism, *β*‐oxidation breaks down the long carbon chains of fatty acids into acetyl‐CoA and then sends them to the TCA cycle [69, 70]. These processes take place in the mitochondria. Given that cuproptosis is dependent on mitochondrial stress and the binding of Cu to lipoylated components of the TCA cycle [26, 27], combined with our results, it is reasonable to believe that tumors with high fatty acid metabolism are equally sensitive to cuproptosis. This may contribute to the development of individualized cuproptosis therapy.

Strikingly, the present study revealed a strong correlation between cuproptosis and immune‐related pathways and signatures in most tumors, suggesting that cuproptosis may be involved in tumor microenvironment remodeling in tumors. High‐cuproptosis activity tends to imply lower immune response and immune cell infiltration. As existing studies have shown that oxidative phosphorylation and TCA cycle metabolism are also crucial in immune cell activation and metabolism [71], we consider that immune cells may be equally sensitive to cuproptosis. Previous studies have shown that pre‐existing antitumor immunity tends to be associated with a better prognosis [72]. However, in our study, although high‐cuproptosis activity reduced the immune response, it still improved the prognosis of a variety of tumors. This may seem contradictory; but numerous studies have also shown that even large amount of immune components does not mean a better prognosis, which is related to various factors, such as the aggressiveness of the tumor itself, the composition of the tumor microenvironment, and the relative predominance of immune stimulatory and suppressive factors [42, 73, 74]. Thus, better prognosis in high‐cuproptosis tumors may be associated with low stromal infiltration and low immunosuppression. This may also be one of the reasons why patients with high cuproptosis in the immunotherapy cohort had better outcomes. In the immunotherapy cohorts, although cuproptosis activity was negatively or not correlated with immune infiltration and PD‐(L)1 expression, patients with high cuproptosis still had good outcomes, possibly due to the lower stromal infiltration. In fact, PD‐(L)1 expression in recent studies has failed to serve as a good predictor of response to ICI therapy [73]. Furthermore, clinical trial‐based studies have demonstrated that TGF‐*β* attenuates tumor response to anti‐PD‐L1 therapy by excluding T cells [42]. The present study revealed that in patients with high‐cuproptosis activity, low TGF‐*β* signaling may account for the positive outcome of patients with high cuproptosis in ICI therapy. However, the specific effects and mechanisms of cuproptosis in immunotherapy need to be elucidated in future basic and clinical studies. Similarly, the main limitation of this manuscript is that the current results are based on a comprehensive analysis of big data, and therefore a large number of experiments are needed to verify the existing results and uncover potential mechanisms.

## CONCLUSION

In conclusion, this study is the first to comprehensively examine the clinical and molecular characteristics of cuproptosis regulators and cuproptosis activity in 33 tumors, providing valuable resources and a reference point for future cuproptosis‐related studies. We showed that cuproptosis is associated with the prognosis of multiple tumors. Moreover, cuproptosis negatively correlates with multiple tumor‐related pathways, including TGF‐*β*/EMT pathway, as well as inflammatory responses, and exhibited potential to predict the outcome of immunotherapy. Cuproptosis may be used to develop a novel strategy for the treatment of cancer.

## METHODS

### SNV and CNV analysis

SNV and CNV data of 33 tumor types from TCGA database were obtained from Xena Functional Genomics Explorer (https://xenabrowser.net/datapages/). The sample size of each tumor type is summarized in Supporting Information Table S12. SNV data retained only nonsilent mutations, including Missense_Mutation, Nonsense_Mutation, Frame_Shift_Del, Splice_Site, Frame_Shift_Ins, In_Frame_Del, and Nonstop_Mutation in this study. According to previous studies [28], values equal to 2 in CNV data are considered as amplifications, and values equal to –2 are considered as deep deletions. SNV and CNV ratios were calculated for each tumor type. In addition, the overall somatic alterations in the OncoPrint plot were generated using the R package “ComplexHeatmap” [75].

### mRNA expression analysis and cuproptosis scores

Normal tissue expression data from the Genotype‐Tissue Expression data set was obtained from Xena to compare the expression differences of cuproptosis regulators in different normal tissues of healthy individuals. In addition, gene expression data for 11,060 tumor patient samples from the TCGA data set was also obtained from Xena. This data set was generated by the TCGA PanCan Atlas project. The gene expression data have been normalized for batch effects and the expression data were log2(*x* + 1) transformed. The interaction network between the cuproptosis regulators was constructed using the STRING database (https://string-db.org/). Out of the 33 tumor types, only 17 tumor types containing more than five pairs of tumor and normal samples were included in the differential expression analysis between tumor and normal samples. The fold change was calculated as the ratio of the mean of the tumor sample expression to the mean of the normal sample expression, and the *p* value was obtained using the *t* test. On the basis of the mRNA expression levels of cuproptosis regulators, potential cuproptosis‐related clusters were identified via unsupervised consensus clustering based on the PAM algorithm. A total of 1000 bootstrap runs were performed, with each bootstrap including 80% of the patients and the number of clusters set to 2–10. The consensus cumulative distribution function and delta area were used to define the optimal number of clusters [76].

To understand the overall cuproptosis level of each sample, we used a previously reported method [41, 77, 78, 79]. The GSVA algorithm was used to calculate the gene set enrichment scores for positive and negative regulators of cuproptosis, which were defined as the cuproptosis‐positive score and cuproptosis‐negative score, respectively, and the cuproptosis activity score was defined as the difference between them. On the basis of the cuproptosis activity score, we identified cuproptosis phenotype‐related genes by the Spearman correlation analysis. Only genes with the absolute values of correlation coefficients >0.3 and *p* < 0.001 were included. Subsequently, GO enrichment analysis and KEGG pathway analyses were performed using the R package “clusterProfiler.”

### Methylation analysis

DNA methylation data for TCGA samples (Methylation450K) were obtained from Xena, and only those probes that mapped to the promoter region of the cuproptosis regulator were used for subsequent analysis. For genes containing multiple probes, the mean *β* value of all probes was used as the methylation level. Only 16 tumor types with at least five tumor–normal pairs were retained in the differential methylation analysis, and fold changes and *p* values were calculated using the same method as that used for differential expression analysis. Subsequently, after integrating the methylation and gene expression data of cuproptosis regulators, correlations and *p* values were determined using Spearman's correlation analysis.

After combining the methylation data of cuproptosis regulators and clinical information, the hazard ratio of regulator methylation was calculated based on Cox regression analysis. A Hazard ratio >1 represented high methylation associated with poor OS, which indicates a high risk. Each tumor type was divided into two groups based on the median methylation level of each regulator and a log‐rank test was performed to calculate the *p* value.

### MiRNAs, lncRNAs, and TFs analyses

The normalized miRNA expression data of TCGA samples were downloaded from Xena, and the batch effect was corrected. The miRNA regulatory data of cuproptosis regulators were collected from databases, including experimentally validated (miRTarBase v9.0 and TarBase v8.0) and predicted (Targetscan v8.0) miRNA–mRNA pairs. Subsequently, the miRNA and mRNA expression data from TCGA were integrated, and for each tumor type, correlations were calculated separately for each miRNA–mRNA pair using Spearman's correlation analysis. Only miRNA–mRNA pairs with correlation coefficients <–0.2 and *p* < 0.05 were considered as potential regulatory pairs. The miRNA regulatory network was constructed using Cytoscape_v3.8.2.

The lncRNA and TF regulatory data were obtained from a previous pan‐cancer study [34]. Using these data, we screened potential lncRNA–mRNA pairs and TF–mRNA pairs targeting cuproptosis regulators in each tumor and calculated the expression correlation of each lncRNA–mRNA and TF–mRNA pair using Spearman's correlation analysis for each tumor type. Only lncRNA–mRNA pairs with absolute values of correlation coefficients >0.2 and *p* < 0.05, and TF–mRNA pairs with absolute values of correlation coefficients >0.3 and *p* < 0.05, were retained. The lncRNA and TF regulatory networks were constructed using Cytoscape_v3.8.2.

### Survival analysis

Survival analysis was performed in Sangerbox (http://vip.sangerbox.com/home.html) [80]. Patient survival status and clinical information can be found in Supporting Information Table S13. After integration of cuproptosis regulators' expression data, cuproptosis scores, and clinical information, hazard ratios were calculated for each variable relative to OS, DFI, and PFI for each tumor type based on Cox regression analysis to determine high or low risk. Each tumor was divided into two groups using the median of each variable as the cutoff value, and a log‐rank test was performed to determine the *p* values. For OS, the optimal cutoff value was used to divide the tumors into two groups to plot the Kaplan–Meier survival curves and perform log‐rank tests. In addition, we obtained an LGG cohort from the CGGA database from GlioVis (http://gliovis.bioinfo.cnio.es/) to validate the prognostic value of cuproptosis regulators and scores.

### Pathway activity analyses

The cancer hallmark gene sets were downloaded from the Molecular Signatures Database (MSigDB, https://www.gsea-msigdb.org/gsea/msigdb/), and the GVSA algorithm was used to infer the hallmark pathway scores for all tumor samples in TCGA. We obtained the latest gene set of cellular senescence from a recent study of Saul et al. [81]. The cell cycle gene set was also obtained from the MSigDB database. The correlation between the cuproptosis activity score and each pathway/marker was subsequently calculated for each tumor type using Spearman's correlation analysis. In addition, the mutation burden, HRD score, loss of heterozygosity (LOH) score, large‐scale state transitions (LST) score, and telomeric allelic imbalance (TAI) score were collected from a previous study [82]. HRD scores were the sum of LOH, LST, and TAI scores.

### Immune characteristic analysis

The ImmuneScore (representing the overall level of immune cell infiltration), StromalScore (representing the overall level of stromal infiltration), and ESTIMATEScore (negatively correlated with tumor purity) were inferred for each tumor sample using the ESTIMATE algorithm [83], and the correlation between the cuproptosis activity score and these three were calculated for each tumor type using Spearman's correlation analysis. The abundance of 22 immune cells calculated based on the CIBERSORT algorithm was collected from a previous publication [37], and the correlation between immune cell abundance and cuproptosis activity was calculated for each tumor type. In addition, immune activation‐related genes, immune checkpoint‐related genes, and TGF‐*β*/EMT pathway‐related genes were collected from a previous publication by Zeng et al. [38].

For the immunotherapy datasets, gene expression data and clinical information for the IMvigor210 cohort were obtained from IMvigor210 Core Biologies (http://research-pub.gene.com/IMvigor210CoreBiologies) [42]. Gene expression data were converted to transcripts per kilobase million values and log2(*x* + 1) transformation was performed. Normalized gene expression data and clinical information for the GSE78220 cohort were obtained from the Gene Expression Omnibus database (https://www.ncbi.nlm.nih.gov/geo/) [41], and gene expression data were transformed using log2(*x* + 1).

### Drug sensitivity analysis

Normalized gene expression data of 809 tumor cell lines and response data for each cell line to 198 compounds were downloaded from the GDSC database [84], and the drug response data were converted to the IC_50_. Spearman's correlations between cuproptosis activity and IC_50_ for each drug were subsequently calculated, and only drugs with absolute values of correlation coefficients >0.1 and *p* < 0.05 were considered to be associated with cuproptosis. The target data of the drugs were then matched with those of screened drugs. In addition, we estimated the IC_50_ of every drug in individual ACC patient based on oncoPredict algorithm using the gene expression profile of these cell lines and drug response data as the training set.

Methods containing more details can be found in the Supplementary protocols.

## AUTHOR CONTRIBUTIONS

Changwu Wu conceived the study, performed the literature search and bioinformatics analysis, prepared and revised the figures and manuscript. Jun Tan, Chaoying Qin, Yuzhe Li, and Yimin Pan helped with data collection, analysis, and interpretation. Yimin Pan, Xiangyu Wang, and Wenyong Long analyzed data and revised the manuscript. Qing Liu helped conceive this research and revise the manuscript. All authors read and approved the final manuscript.

## CONFLICT OF INTEREST

The authors declare no conflict of interest.

## Supporting information

Supporting information.

Supporting information.

## Data Availability

The data generated in this study are available within the article and its supplementary data files. The data analyzed in this study were obtained from the Xena Functional Genomics Explorer (https://xenabrowser.net/datapages/), GlioVis (http://gliovis.bioinfo.cnio.es/), IMvigor210 Core Biologies (http://research-pub.gene.com/IMvigor210CoreBiologies), and GSE78220 (https://www.ncbi.nlm.nih.gov/geo/). The code used in this study can be found in GitHub (https://github.com/Changwuuu/Cuproptosis-pancancer.git).
