# Supplementary material for: Pan‐cancer analyses reveal molecular and clinical characteristics of cuproptosis regulators
Source: Imeta. 2022 Dec 7;2(1):e68. doi: 10.1002/imt2.68 (PMC10989956; doi:10.1002/imt2.68)
Supplement: Supplementary file 1 — Supporting information. [file IMT2-2-e68-s002.docx]

**Supplementary Protocols and Figures**

**Pan-cancer analyses reveal molecular and immunological** **characteristics of cuproptosis**

Changwu Wu^1^, Jun Tan^1^, Xiangyu Wang^1^, Chaoying Qin^1^, Wenyong Long^1^, Yimin Pan^2^, Yuzhe Li^1^, Qing Liu^1,3,*^

1. Department of Neurosurgery, Xiangya Hospital, Central-South University, Changsha, China;

2. Department of Neurosurgery, Devision of Experimental Neurosurgery, University of Heidelberg, Heidelberg, Germany

3. Institute of Skull Base Surgery and Neuro-oncology at Hunan, Changsha, China.

* Corresponding author: *Qing Liu, M.D. & Ph.D., Professor and Vice Chairman, Department of Neurosurgery, Xiangya Hospital, Central South University, Institute of Skull Base Surgery & Neuro-oncology at Hunan, 87 Xiangya Road, Changsha, Hunan, 410008, P.R. China E-mail: liuqingdr@csu.edu.cn*

**Supplementary Protocols**

**SNV and CNV analysis**

The analysis of SNV and CNV was performed according to the following steps: (1) The SNV data (mc3.v0.2.8.PUBLIC file), CNV data (TCGA.PANCAN. CopyNumber_Gistic2_all_thresholded.by_genes file) and clinical data of the TCGA cohort were downloaded from Xena Functional Genomics Explorer (<https://xenabrowser.net/datapages/>, accessed on March 2022). After matching, 10680 samples with both SNV, CNV and tumor type information were screened. The sample size of each tumor type was summarized in Supplementary Table S1; (2) Extracing information of cuproptosis regulators from SNV and CNV data. SNV data retained only non-silent mutations, including Missense_Mutation, Nonsense_Mutation, Frame_Shift_Del, Splice_Site, Frame_Shift_Ins, In_Frame_Del, and Nonstop_Mutation; (3) According to previous studies [1], values equal to 2 in CNV data were considered as amplifications and values equal to –2 are considered as deep deletions; (4) Sorting out SNV and CNV information of cuproptosis regulators in Excel, then calculate SNV and CNV frequencies for each tumor type using the formula: Number of SNV or CNV Samples/Number of Cancer Samples; (5) After importing SNV and CNV data into R software, use R package “ComplexHeatmap” to generate OncoPrint plots and heatmaps. The relevant code can be found in GitHub (https://github.com/Changwuuu/Cuproptosis-pancancer.git).

**mRNA expression analysis and cuproptosis scores**

The analysis of mRNA expression was performed according to the following steps: (1) Normal tissue expression data (gtex_RSEM_Hugo_norm_count file) and phenotype information from the Genotype-Tissue Expression dataset were obtained from Xena (accessed on March 2022), containing a total of 7823 samples from 28 different tissues. Then we extracted the mRNA expression of 10 cuproptosis regulators to compare the expression differences in different normal tissues and plotted the heatmap in Excel; (2) Gene expression data (EB++AdjustPANCAN_IlluminaHiSeq_RNASeqV2.geneExp file) for 11060 tumor patient samples from the TCGA dataset was obtained from Xena (accessed on March 2022). This dataset was generated by the TCGA PanCan Atlas project. For comparison across all TCGA samples, it has been normalized for batch effects using a novel algorithm called EB++ and the expression data was log2(x+1) transformed. Specific details of the correction of batch effects can be found in the publication of Hoadley et al [2]. A large number of high-quality studies used this dataset (<https://www.cell.com/pb-assets/consortium/pancanceratlas/pancani3/index.html>); (3) The interaction network between the cuproptosis regulators was constructed using the STRING database (<https://string-db.org/>). After entering the cuproptosis regulator, we used the default parameters (medium confidence, full STRING network) to build the PPI network; (4) For differential expression analysis between tumor tissue and normal tissue, only 17 tumor types containing more than five pairs of tumor and normal samples were included. The fold change was calculated as the ratio of the mean of the tumor sample expression to the mean of the normal sample expression, and the *P*-value was obtained using the t-test; (5) For cluster analysis, we first extracted the mRNA expression data of 10 cuproptosis regulators from all tumor samples and constructed an expression matrix. Based on this, we performed an unsupervised consensus clustering analysis using the PAM algorithm based on the R package “ConsensusClusterPlus” to identify potential cuproptosis-related clusters. A total of 1 000 bootstrap runs were performed, with each bootstrap including 80% of the patients and the number of clusters set to 2–10. The consensus cumulative distribution function and delta area were used to define the optimal number of clusters [3]. Clinical information and clusters were then matched by barcode for each sample. Comparing survival differences between cluster1 and cluster2 using the single-variable survival analysis tool in Sangebox (<http://sangerbox.com/Tool>) [4].

For the generation of cuproptosis scores, we used a previously reported method [5-8]. Firstly, we constructed a “gmt” file containing gene sets of cuproptosis positive regulators and negative regulators. Subsequently, the gene set enrichment scores of cuproptosis positive regulators and negative regulators were calculated using GSVA algorithm based on the mRNA expression of all samples. The gene set enrichment scores of cuproptosis positive regulators and negative regulators were defined as the cuproptosis positive score and cuproptosis negative score, respectively. Then the cuproptosis activity score was caculated using the formula:

Cuproptosis activity score = Cuproptosis positive score - Cuproptosis negative score

For the identification of cuproptosis phenotype-related genes, we performed the spearman correlation analysis between cuproptosis activity score and all genes. Only genes with the absolute values of correlation coefficients > 0. 3 and *P* < 0. 001 were defined as cuproptosis-related genes. Subsequently, GO enrichment analysis and KEGG pathway analysis of cuproptosis--related genes were performed using the R package “clusterProfiler”. We extracted the top 10 terms from GO enrichment analysis and the top 30 terms from KEGG pathway analysis for visualization respectively.

**Methylation analysis**

DNA methylation data (PANCAN_HumanMethylation450.betaValue file) for TCGA samples (Methylation450K, n = 9 639) were obtained from Xena (accessed on March 2022). We extracted all the probes corresponding to the cuproptosis regulator based on the probe map, and subsequently screened the probes corresponding to the promoter region of the cuproptosis regulator by MEXPRESS (<https://mexpress.be/>). Only those probes that mapped to the promoter region of the cuproptosis regulator were used for subsequent analysis. After extracting the methylation β values corresponding to these probes, the mean β value of the promoter region probes of single cuproptosis regulator was used as the methylation level. The methylation levels of the cuproptosis regulators were then matched to the clinical data based on the barcode of each sample.

For the differential methylation analysis, only 16 tumor types with at least five tumor–normal pairs were retained. Then the fold changes and *P*-values were calculated using the same method as that used for differential mRNA expression analysis. Subsequently, after integrating the methylation and gene expression data of cuproptosis regulators, correlations and p-values were determined using Spearman’s correlation analysis for each tumor type. After extracting significant results, visualize them using the bubble plotting tool in Sangerbox. After combining the methylation data of cuproptosis regulators and survival data, the hazard ratio of regulator methylation was calculated based on the Cox regression analysis in Sangerbox. Hazard ratio > 1 represented high methylation associated with poor OS, which indicates a high risk. Each tumor type was divided into two groups based on the median methylation level of each regulator and a log-rank test was performed to calculate the *P*-value.

**miRNA, lncRNA, and TF analyses**

The miRNA expression data (pancanMiRs file) of TCGA samples (n = 10818) were downloaded from Xena (accessed on March 2022). This dataset was also generated by the TCGA PanCan Atlas project. It has been normalized for batch effects using a novel algorithm called EB++ and the expression data was log2(x+1) transformed by Hoadley et al [2]. Subsequently, miRNA and mRNA expression data from TCGA were integrated according to the barcode of each sample.

The miRNA regulatory data of cuproptosis regulators were collected from databases, including experimentally validated and predicted miRNA–mRNA pairs. The experimentally validated miRNA–mRNA pairs were extracted from miRTarBase v9.0 (<https://mirtarbase.cuhk.edu.cn/>, accessed on March 2022) and TarBase v8.0 (<https://dianalab.e-ce.uth.gr/html/diana/web/index.php?r=tarbasev8/index>, accessed on March 2022). The predicted miRNA–mRNA pairs were extracted from Targetscan v8.0 (<https://www.targetscan.org/vert_80/>, accessed on March 2022). All three databases are easy to use, each cuproptosis regulator was entered to display the paired miRNAs. Then we merged the results of the three databases to obtain the list of miRNA-mRNA pairs for each cuproptosis regulator. For each tumor type, correlations were calculated separately for each miRNA–mRNA pair using Spearman’s correlation analysis. Only miRNA–mRNA pairs with correlation coefficients < –0.2 and *P* < 0.05 were considered as potential regulatory pairs. After calculation, we obtained miRNA-mRNA pairs for each cuproptosis regulator in all tumor types. Subsequently, miRNA-mRNA pairs that appeared in at least five tumor types and targeted at least two cuproptosis regulators were screened in Excel and made into a list. Finally, the miRNA-mRNA list was imported into Cytoscape_v3.8.2 to construct and visualize the miRNA regulatory network.

The lncRNA and TF regulatory data were obtained from a previous pan-cancer study [9]. Using this data, we screened potential lncRNA–mRNA pairs and TF–mRNA pairs targeting cuproptosis regulators in each tumor and calculated the expression correlation of each lncRNA–mRNA and TF–mRNA pair using Spearman’s correlation analysis for each tumor type. Only lncRNA–mRNA pairs with absolute values of correlation coefficients > 0.2 and *P* < 0.05, and TF–mRNA pairs with absolute values of correlation coefficients > 0.3 and *P* < 0.05, were retained. LncRNA-mRNA pairs that targeted at least three cuproptosis regulators and TF-mRNA pairs that targeted at least five cuproptosis regulators were screened in Excel and made into two lists. The two lists were imported into Cytoscape_v3.8.2 to construct and visualize the LncRNA and TF regulatory networks.

**Survival analysis**

First, we integrated the cuproptosis regulator expression data, cuproptosis scores, and clinical information. A total of 10058 tumor samples with matched expression data and clinical information. We inputted survival data of each tumor type into the bulk survival analysis tool (Using default parameters) of Sangerbox to calculate hazard ratios for each cuproptosis regulator or score relative to OS, DFI and PFI. The calculation principle is based on based on Cox regression analysis. Hazard ratio >1 was considered high risk. Each tumor was divided into two groups using the median of each cuproptosis regulator or score as the cutoff value, and a log-rank test was performed to determine the *P*-values. Subsequently, results with significance were visualized using the bubble plotting tool of Sangerbox. For OS, we also calculated the optimal cutoff value of cuproptosis activity score in each tumor using the Kaplan Meier curve plotting tool of Sangerbox, and used optimal cutoff value to divide the tumors into two groups to plot the Kaplan–Meier survival curves.

To validate the prognostic value of cuproptosis regulators and scores, we obtained a LGG cohort from the Chinese Glioma Genome Atlas (CGGA) from GlioVis (<http://gliovis.bioinfo.cnio.es/>, accessed on August 2022). The mRNA expression data of this cohort has been normalized in GlioVis and performed log2(x+0.5) transformation. We extracted 431 LGG samples with complete clinical information from this cohort. Then we calculated the cuproptosis scores of this cohort using the same method as described earlier. We matched mRNA expression data of cuproptosis regulators, cuproptosis scores, and clinical information for each sample. Then Hazard ratios for each cuproptosis regulator and score were calculated using Cox regression analysis and plotted a forest plot. Kaplan Meier curves were generated in the single-variable survival analysis tool of Sangebox.

**Pathway activity analyses**

The cancer hallmark gene sets (h.all.v7.5.1.symbols.gmt) were downloaded from the MSigDB (<https://www.gsea-msigdb.org/gsea/msigdb/>, accessed on March 2022), and the GVSA algorithm was used to infer the hallmark pathway enrichment scores for all tumor samples in TCGA. The method is consistent with the generation of cuproptosis scores. We obtained the latest gene set of cellular senescence from a recent study of Saul et al [10]. The cell cycle gene set was also obtained from the KEGG database, which can be accessed in the MSigDB database. After matching the hallmark pathway score and clinical information, the correlation between the cuproptosis activity score and each hallmark pathway was subsequently calculated for each tumor type using Spearman’s correlation analysis. The correlation coefficients of each tumor type were then visualized using the heat map plotting tool of Sangerbox. In addition, the mutation burden, HRD score, loss of heterozygosity (LOH) score, large-scale state transitions (LST) score, and telomeric allelic imbalance (TAI) score were collected from a previous study [11]. HRD score was the sum of LOH, LST, and TAI scores. After matching cuproptosis activity score, HRD, LOH, LST, and TAI scores by barcode for each sample, we calculated the correlation using Spearman’s correlation analysis and visualized the significant results in the bubble plotting tool. In addition, we calculated the correlation between the cuproptosis activity score and cell senescence/cycle markers in each tumor type using Spearman’s correlation analysis and visualized the correlation coefficients using the heatmap plotting tool of Sangerbox.

**Immune characteristic analysis**

The ImmuneScore (representing the overall level of immune cell infiltration), StromalScore (representing the overall level of stromal infiltration), and ESTIMATEScore (negatively correlated with tumor purity) were inferred for each tumor sample using the ESTIMATE algorithm after inputting the mRNA expression data into R software [12], and the correlation between the cuproptosis activity score and these three were calculated for each tumor type using Spearman’s correlation analysis. The bubble plotting tool was used for visualization. The abundance of 22 immune cells calculated based on the CIBERSORT algorithm was collected from a previous publication [13]. Subsequently, we matched the cuproptosis activity socre to the obtained immune cell infiltration data. The correlation between immune cell abundance and cuproptosis activity was calculated for each tumor type using Spearman’s correlation analysis. In addition, immune activation-related genes, immune checkpoint-related genes, and TGFβ/EMT pathway-related genes were collected from a previous publication by Zeng et al [14]. We extracted the expression data of these genes from the mRNA expression matrix. Then we matched the expression data and cuproptosis activity score and calculated the Spearman’s correlation between them. In addition, we obtained the pan-cancer immune subtype classification data from the study of Thorsson et al [13]. We compared the difference in cuproptosis activity score among these immune subtypes after matching the immune subtype and cuproptosis activity score for each sample by barcode.

For the immunotherapy datasets, gene expression data and clinical information for the IMvigor210 cohort were obtained from IMvigor210 Core Biologies (<http://research-pub.gene.com/IMvigor210CoreBiologies>, accessed on March 2022) [15], and this link provides details on how to use the IMvigor210 cohort. Gene expression data of IMvigor210 cohort were converted to transcripts per kilobase million values and log2(x +1) transformation was performed. Calculating cuproptosis score by GSVA algorithm, and Calculating ImmuneScore, StromalScore and ESTIMATEScore by ESTIMATE algorithm. We merged all data and only retained samples with complete survival information and treatment response information. Then, we calculated the Spearman’s correlation between cuproptosis activity score and PL-1 expression and plotted the Kaplan Meier survival curve of cuproptosis activity score using the single-variable survival analysis tool in Sangebox. In addition, normalized gene expression data and clinical information for the GSE78220 cohort were obtained from the Gene Expression Omnibus database (<https://www.ncbi.nlm.nih.gov/geo/>, accessed on March 2022) [6], and gene expression data were transformed using log2(x+1). We used the same method as the IMvigor210 cohort to analyze the GSE78220 cohort.

**Drug sensitivity analysis**

Normalized gene expression data of 809 tumor cell lines and response data for each cell line to 198 compounds were downloaded from the Genomics of Drug Sensitivity in Cancer database (GDSC, <https://www.cancerrxgene.org/>, accessed on March 2022) [16], and the drug response data were converted to IC_50_ values. The cuproptosis scores were calculated for each cell line using the GSVA algorithm. After matching the cuproptosis activity and IC_50_ values, Spearman’s correlations between cuproptosis activity and IC_50_ value for each drug were subsequently calculated, and only drugs with absolute values of correlation coefficients > 0.1 and *P* < 0.05 were considered to be associated with cuproptosis. The target data of the drugs were then matched with those of screened drugs. We used the bar graph plotting tool of Sangerbox to visualize these results.

In addition, we extracted the mRNA expression data of ACC samples and estimated the IC_50_ of every drug using oncoPredict algorithm [17]. This algorithm is based on ridge regression. We used the gene expression profile of GDSC tumor cell lines and drug response data as the training set to infer the IC_50_ of 198 drugs in individual ACC sample. Then we calculated the correlation between cuproptosis activity and IC_50_ value for each drug, and only drugs with absolute values of correlation coefficients > 0.2 and *P* < 0.05 were considered to be significant. The target data of the drugs were then matched with those of screened drugs.

The cell model figure of cuproptosis was generated by Figdraw.

**References**

1. Beroukhim, Rameen, Craig H. Mermel, Dale Porter, Guo Wei, Soumya Raychaudhuri, Jerry Donovan, Jordi Barretina, et al. 2010. “The landscape of somatic copy-number alteration across human cancers.” *Nature* 463: 899-905. <https://doi.org/10.1038/nature08822>

2. Hoadley, Katherine A., Christina Yau, Toshinori Hinoue, Denise M. Wolf, Alexander J. Lazar, Esther Drill, Ronglai Shen, et al. 2018. “Cell-of-Origin Patterns Dominate the Molecular Classification of 10,000 Tumors from 33 Types of Cancer.” *Cell* 173: 291-304.e296. https://doi.org/10.1016/j.cell.2018.03.022

3. Wilkerson, Matthew D., D. Neil Hayes. 2010. “ConsensusClusterPlus: a class discovery tool with confidence assessments and item tracking.” *Bioinformatics* 26: 1572-1573. <https://doi.org/10.1093/bioinformatics/btq170>

4. Shen, Weitao, Ziguang Song, Xiao Zhong, Mei Huang, Danting Shen, Pingping Gao, Xiaoqian Qian, et al. 2022. “Sangerbox: A comprehensive, interaction-friendly clinical bioinformatics analysis platform.” *iMeta* 1: e36. https://doi.org/10.1002/imt2.36

5. Hänzelmann, Sonja, Robert Castelo, Justin Guinney. 2013. “GSVA: gene set variation analysis for microarray and RNA-Seq data.” *BMC Bioinformatics* 14: 7. <https://doi.org/10.1186/1471-2105-14-7>

6. Hugo, Willy, Jesse M. Zaretsky, Lu Sun, Chunying Song, Blanca Homet Moreno, Siwen Hu-Lieskovan, Beata Berent-Maoz, et al. 2017. “Genomic and Transcriptomic Features of Response to Anti-PD-1 Therapy in Metastatic Melanoma.” *Cell* 168: 542. <https://doi.org/10.1016/j.cell.2017.01.010>

7. Bailey, Peter, David K. Chang, Katia Nones, Amber L. Johns, Ann-Marie Patch, Marie-Claude Gingras, David K. Miller, et al. 2016. “Genomic analyses identify molecular subtypes of pancreatic cancer.” *Nature* 531: 47-52. <https://doi.org/10.1038/nature16965>

8. Ye, Youqiong, Qingsong Hu, Hu Chen, Ke Liang, Yuan Yuan, Yu Xiang, Hang Ruan, et al. 2019. “Characterization of hypoxia-associated molecular features to aid hypoxia-targeted therapy.” *Nature Metabolism* 1: 431-444. <https://doi.org/10.1038/s42255-019-0045-8>

9. Chiu, Hua-Sheng, Sonal Somvanshi, Ektaben Patel, Ting-Wen Chen, Vivek P. Singh, Barry Zorman, Sagar L. Patil, et al. 2018. “Pan-Cancer Analysis of lncRNA Regulation Supports Their Targeting of Cancer Genes in Each Tumor Context.” *Cell Reports* 23: 297-312.e212. <https://doi.org/10.1016/j.celrep.2018.03.064>

10. Saul, Dominik, Robyn Laura Kosinsky, Elizabeth J. Atkinson, Madison L. Doolittle, Xu Zhang, Nathan K. LeBrasseur, Robert J. Pignolo, et al. 2022. “A new gene set identifies senescent cells and predicts senescence-associated pathways across tissues.” *Nature Communications* 13: 4827. https://doi.org/10.1038/s41467-022-32552-1

11. Knijnenburg, Theo A., Linghua Wang, Michael T. Zimmermann, Nyasha Chambwe, Galen F. Gao, Andrew D. Cherniack, Huihui Fan, et al. 2018. “Genomic and Molecular Landscape of DNA Damage Repair Deficiency across The Cancer Genome Atlas.” *Cell Reports* 23: 239-254.e236. <https://doi.org/10.1016/j.celrep.2018.03.076>

12. Yoshihara, Kosuke, Maria Shahmoradgoli, Emmanuel Martínez, Rahulsimham Vegesna, Hoon Kim, Wandaliz Torres-Garcia, Victor Treviño, et al. 2013. “Inferring tumour purity and stromal and immune cell admixture from expression data.” *Nature Communications* 4: 2612. <https://doi.org/10.1038/ncomms3612>

13. Thorsson, Vésteinn, David L. Gibbs, Scott D. Brown, Denise Wolf, Dante S. Bortone, Tai-Hsien Ou Yang, Eduard Porta-Pardo, et al. 2018. “The Immune Landscape of Cancer.” *Immunity* 48: 812-830.e814. <https://doi.org/10.1016/j.immuni.2018.03.023>

14. Zeng, Dongqiang, Meiyi Li, Rui Zhou, Jingwen Zhang, Huiying Sun, Min Shi, Jianping Bin, Yulin Liao, Jinjun Rao, Wangjun Liao. 2019. “Tumor Microenvironment Characterization in Gastric Cancer Identifies Prognostic and Immunotherapeutically Relevant Gene Signatures.” *Cancer Immunology Research* 7: 737-750. <https://doi.org/10.1158/2326-6066.Cir-18-0436>

15. Mariathasan, Sanjeev, Shannon J. Turley, Dorothee Nickles, Alessandra Castiglioni, Kobe Yuen, Yulei Wang, Edward E. Kadel Iii, et al. 2018. “TGFβ attenuates tumour response to PD-L1 blockade by contributing to exclusion of T cells.” *Nature* 554: 544-548. <https://doi.org/10.1038/nature25501>

16. Yang, Wanjuan, Jorge Soares, Patricia Greninger, Elena J. Edelman, Howard Lightfoot, Simon Forbes, Nidhi Bindal, et al. 2012. “Genomics of Drug Sensitivity in Cancer (GDSC): a resource for therapeutic biomarker discovery in cancer cells.” *Nucleic Acids Research* 41: D955-D961. <https://doi.org/10.1093/nar/gks1111>

17. Maeser, Danielle, Robert F Gruener, Rong Stephanie Huang. 2021. “oncoPredict: an R package for predicting in vivo or cancer patient drug response and biomarkers from cell line screening data.” *Briefings in Bioinformatics* 22. https://doi.org/10.1093/bib/bbab260

**Supplementary Figures**


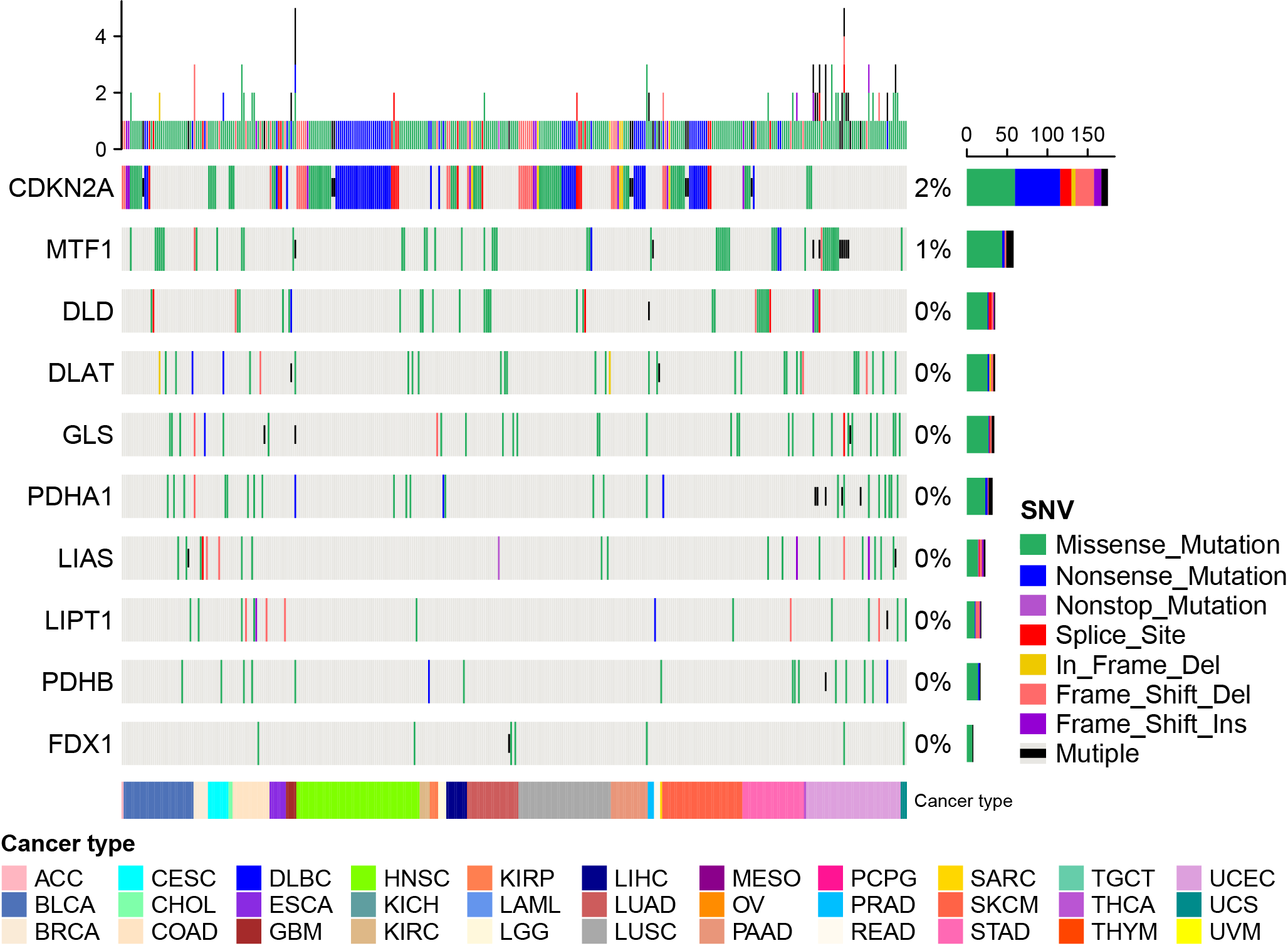


**Figure S1. The SNV landscape of cuproptosis regulators in cancer.** Each row represents a gene, and each column represents a patient. The frequencies of SNV of 10 cuproptosis regulators are presented. Only patients with SNV in the cuproptosis regulators are shown. Alteration rate of each gene is displayed in the right labels.


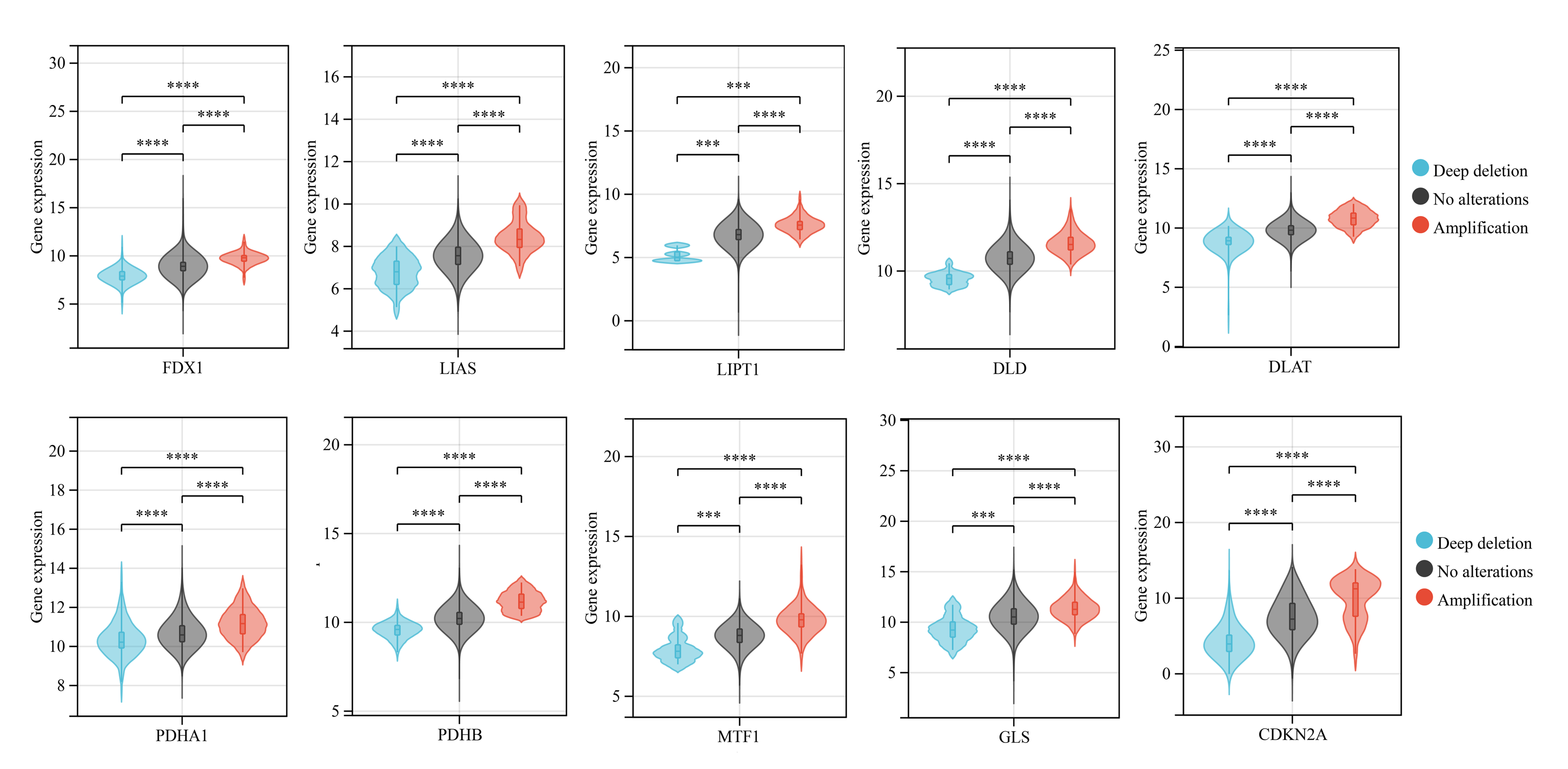


**Figure S2. The relationship between CNV and cuproptosis regulators expression.** Boxplots showing expression of individual cuproptosis regulators in patient samples with no alteration, amplification and deep deletion of correspondent regulator. The differences between any two groups were tested by Student’s t test. *** *P*< 0.001, **** *P*< 0.0001


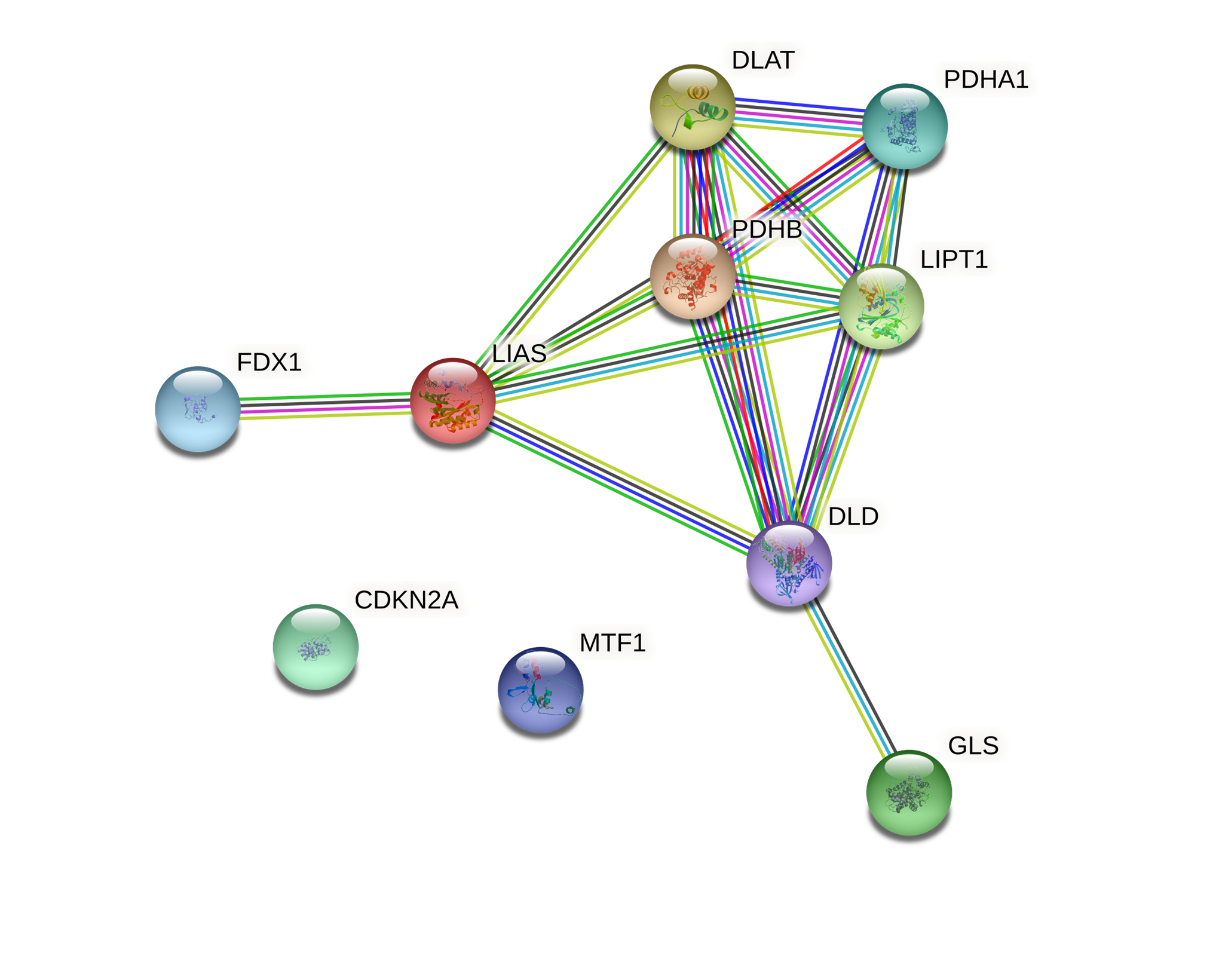
 **Figure S3.** **The interaction relationships among cuproptosis regulators.** An interaction network among cuproptosis regulators based on String database.


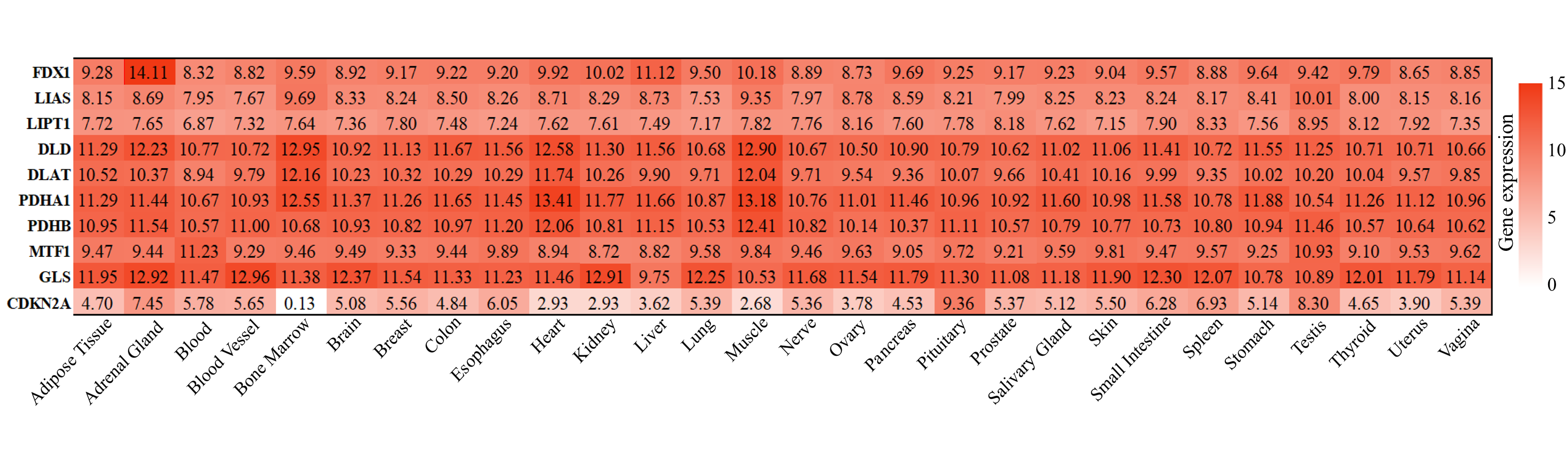


**Figure S4.** **Expression of cuproptosis regulators in normal tissues.** The heatmap showing cuproptosis regulator expression in 28 normal tissues. The intensity of red is proportional to the expression. Each row represents a cuproptosis regulator, and each column is a normal tissue.


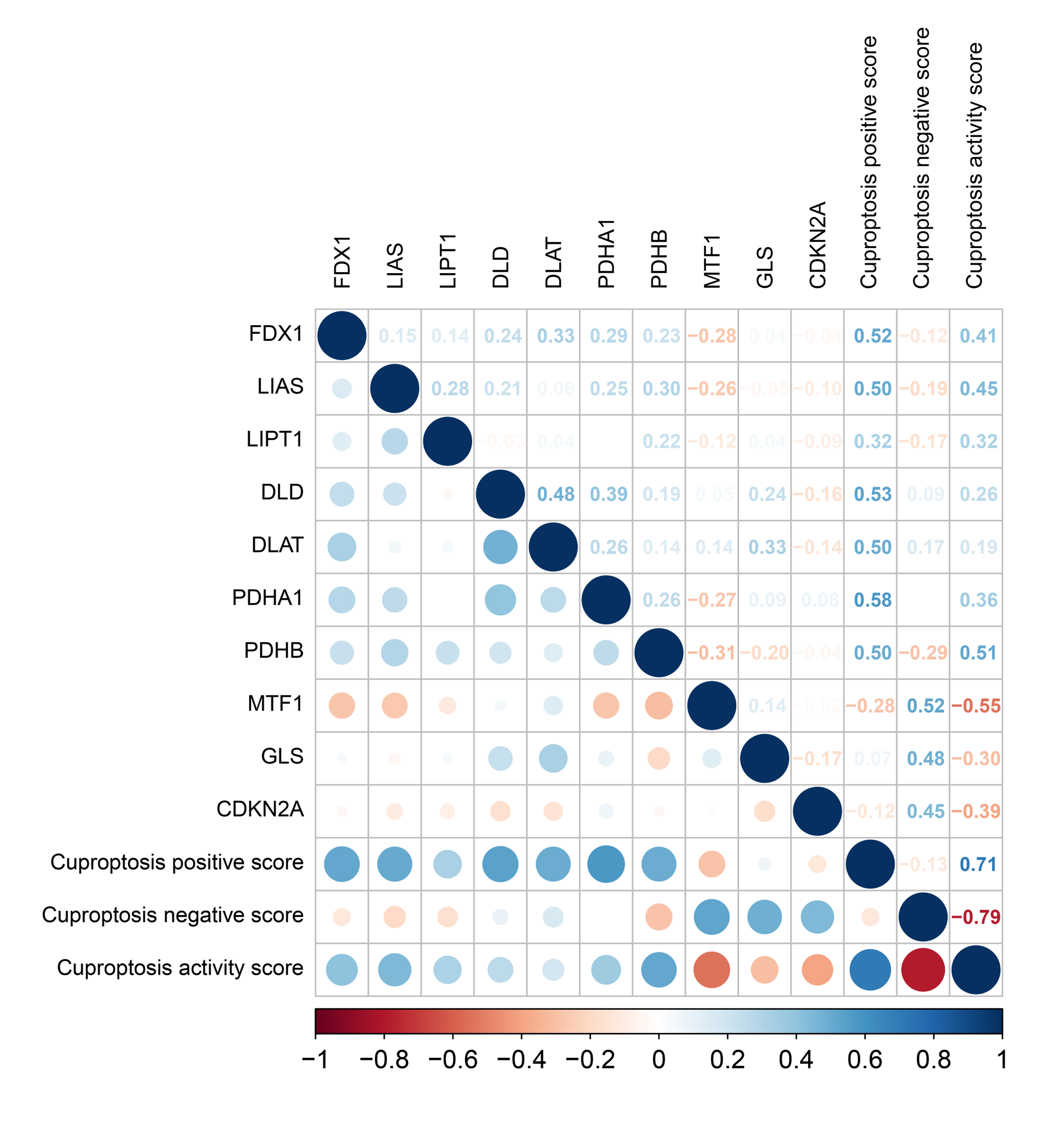


**Figure S5.** **Correlation between cuproptosis regulators/scores.** Correlations between cuproptosis regulators, cuproptosis positive score, cuproptosis negative score, and cuproptosis activity score. Blue represents a positive correlation and red represents a negative correlation. The size of the points represents the size of the absolute value of correlation coefficient. Only points with correlation *P* < 0.05 are shown.


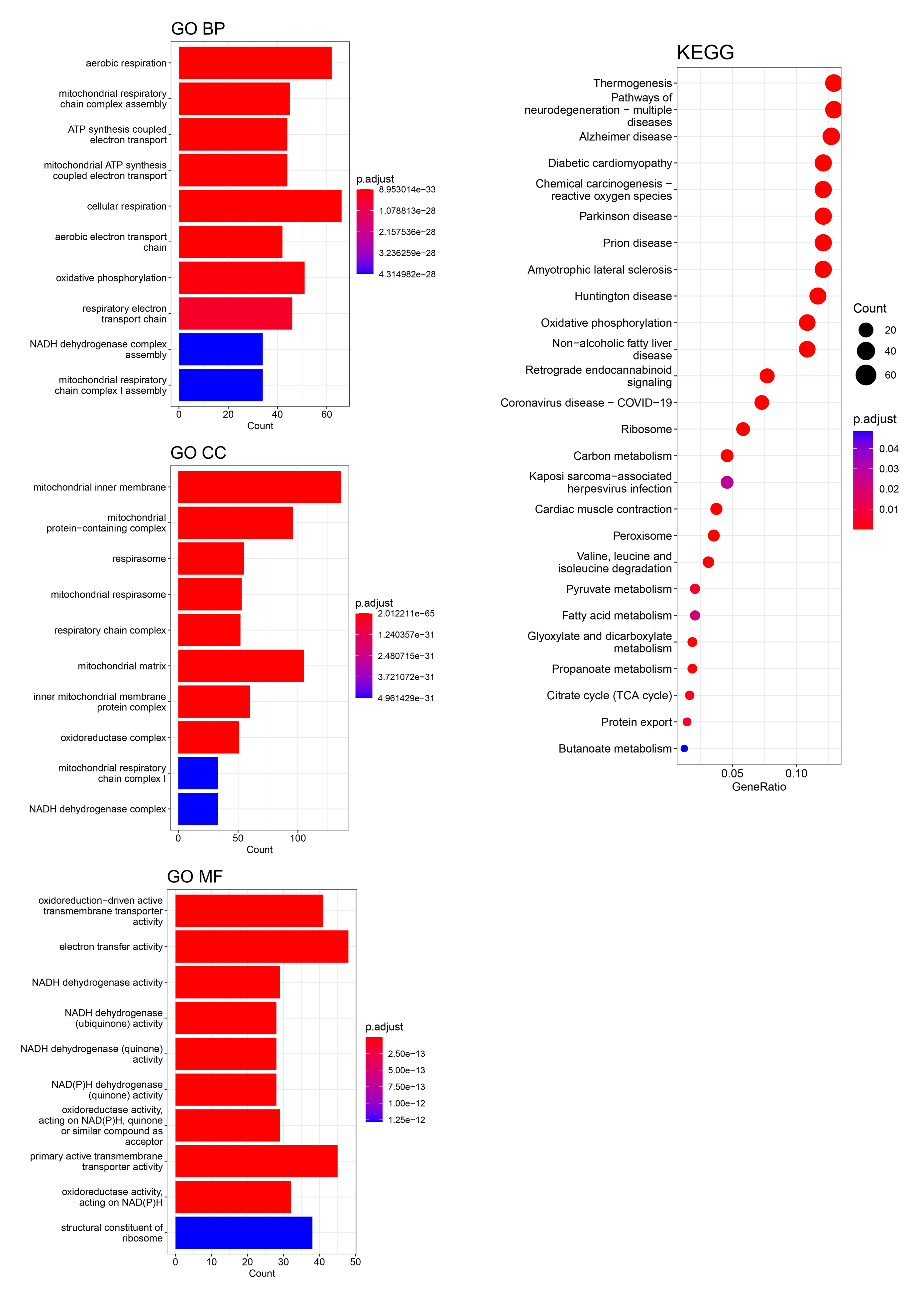


**Figure S6. GO enrichment analysis and KEGG pathway analysis of cuproptosis-related genes.** GO enrichment analysis shows the top 10 terms and KEGG pathway analysis shows the top 30 terms.


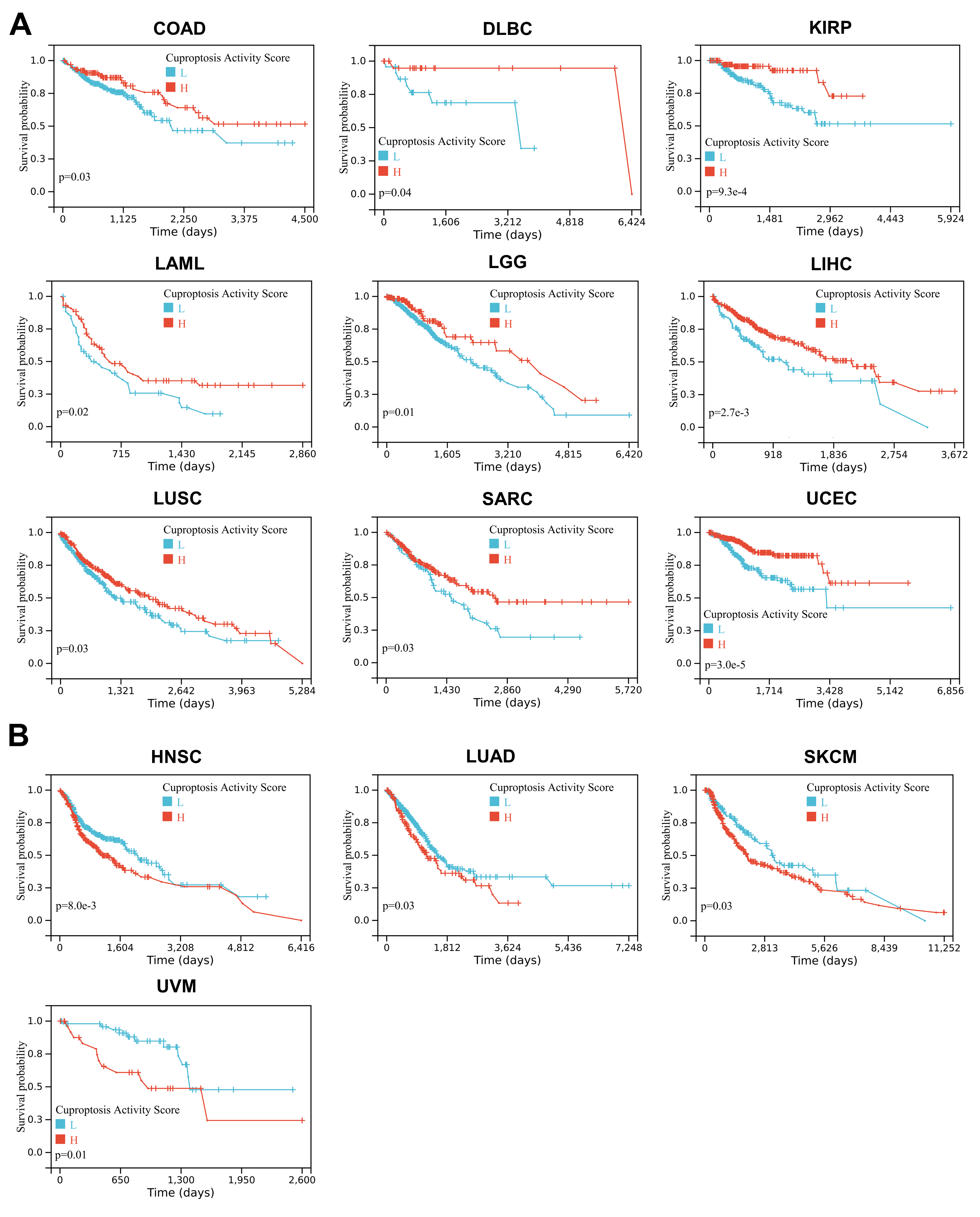


**Figure S7.** **Cuproptosis activity is a prognostic factor in 13 cancer types.** High cuproptosis activity score is associated with both better (A) and worse (B) OS. OS of patients with high cuproptosis activity score was compared with those with low cuproptosis activity score in a Kaplan–Meier survival curve analysis. Statistical significance was assessed by log-rank test.


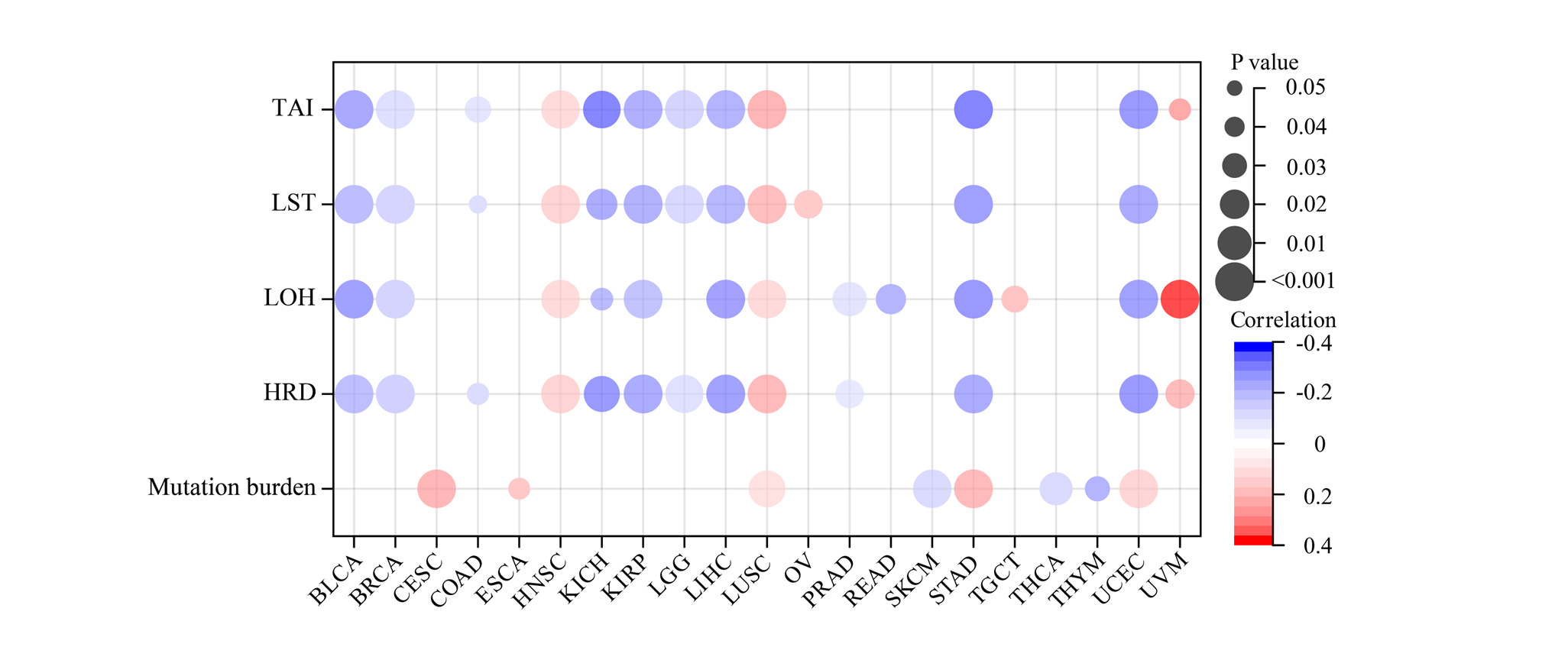


**Figure S8.** **Cuproptosis activity is associated with genomic instability.** Correlation between cuproptosis activity and mutation burden, HRD score, LOH score, LST score and TAI score. Blue points represent a negative correlation and red points represent a positive correlation, where the darker of color, the higher the correlation.


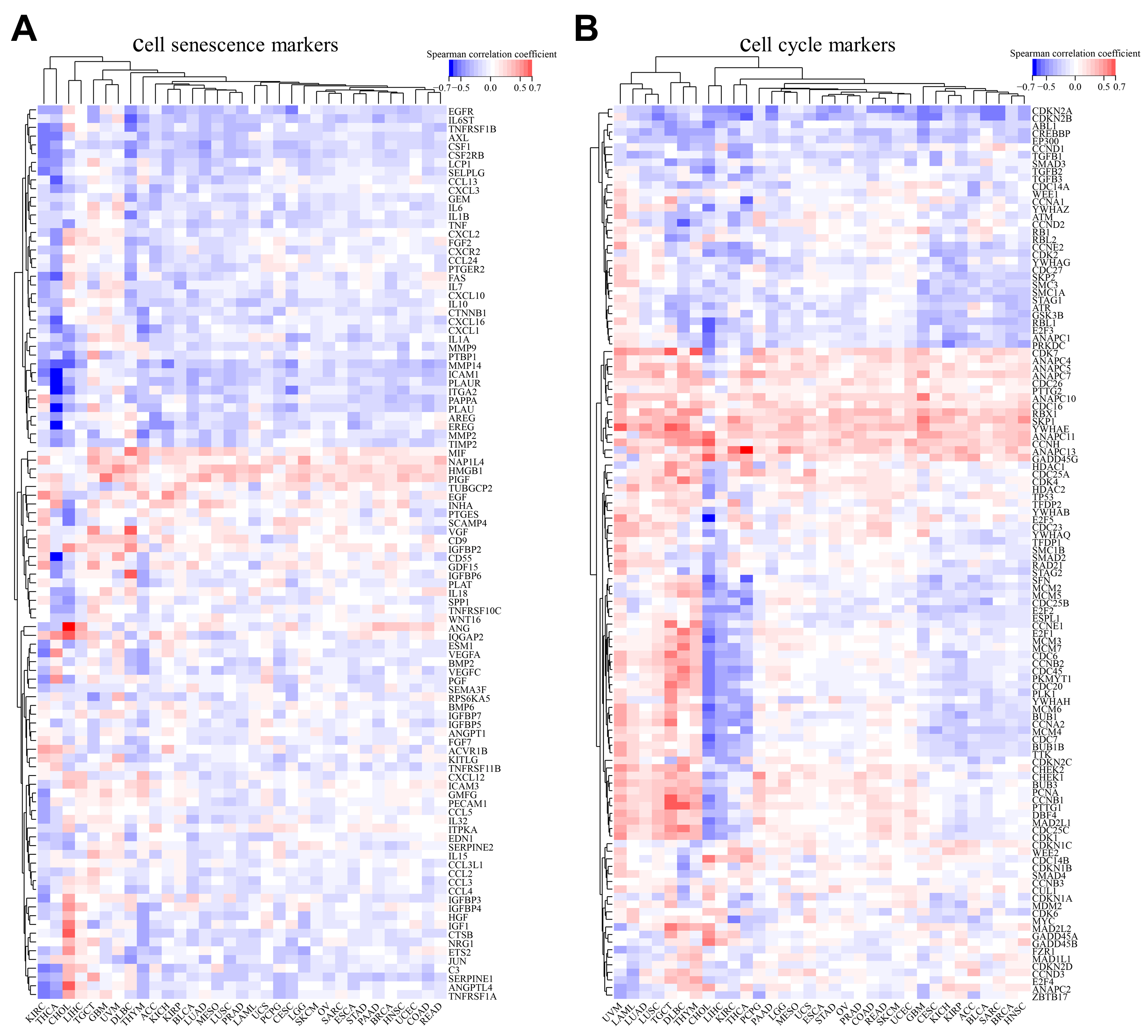


**Figure S9.** **Cuproptosis activity is associated with c****ell senescence and cell cycle markers.** The heatmaps showing the correlation between cuproptosis activity and cell senescence (A) and cell cycle (B) markers in each cancer type. Each column is a cancer type, and each row is a marker. Red represents positive correlation, blue represents negative correlation. Unsupervised clustering used Euclidean distance metric with complete linkage.
